# The charge transport properties of dicyanomethylene-functionalised violanthrone derivatives

**DOI:** 10.3762/bjoc.20.244

**Published:** 2024-11-13

**Authors:** Sondos A J Almahmoud, Joseph Cameron, Dylan Wilkinson, Michele Cariello, Claire Wilson, Alan A Wiles, Peter J Skabara, Graeme Cooke

**Affiliations:** 1 Department of Chemistry, College of Science, Imam Mohammad Ibn Saud Islamic University (IMSIU), Riyadh 11623, Saudi Arabia; 2 School of Chemistry, University of Glasgow, Glasgow, G12 8QQ, UK

**Keywords:** Knoevenagel condensation, organic field-effect transistor, organic semiconductor, violanthrone

## Abstract

The study of organic small molecule semiconductor materials as active components of organic electronic devices continues to attract considerable attention due to the range of advantages these molecules can offer. Here, we report the synthesis of three dicyanomethylene-functionalised violanthrone derivatives (**3a**, **3b** and **3c**) featuring different alkyl substituents. It is found that the introduction of the electron-deficient dicyanomethylene groups significantly improves the optical absorption compared to their previously reported precursors **2a**–**c**. All compounds are p-type semiconductors with low HOMO–LUMO gaps (≈1.25 eV). The hole mobilities, measured from fabricated organic field-effect transistors, range from 3.6 × 10^−6^ to 1.0 × 10^−2^ cm^2^ V^−1^ s^−1^. We found that the compounds featuring linear alkyl chains (**3b** and **3c**) displayed a higher mobility compared to the one with branched alkyl chains, **3a**. This could be the result of the more highly disordered packing arrangement of this molecule in the solid state, induced by the branched side chains that hinder the formation of π–π stacking interactions. The influence of dicyanomethylene groups on the charge transport properties was most clearly observed in compound **3b** which has a 60-fold improvement in mobility compared to **2b**. This study demonstrates that the choice of the solubilising group has a profound effect on the hole mobility on these organic semiconductors.

## Introduction

Recently, organic semiconductors have received considerable attention due to their potential technological applications in semiconductor devices, such as organic field-effect transistors (OFETs) [1–2], organic light-emitting diodes (OLEDs) [3], and organic photovoltaic devices (OPVs)[4–6]. The charge transporting properties of organic semiconductors are key to the success of the devices and research focusing upon increasing this remains an important goal to enhance the commercial viability of the technologies. Typically, organic semiconductor molecules with large fused conjugated systems have achieved high charge carrier mobility. Such molecular structures improve the intermolecular interactions (such as π–π stacking) that are required to facilitate the hopping of charge carriers between adjacent molecules [7–9].

Among many intensively investigated organic semiconductors [10–12], are perylene diimide (PDI) derivatives which feature a rigid, planar, fused π-skeleton. These molecules have been widely utilised as n-type materials, due to their exceptional charge mobility (μ_e_ ≈ 0.1–2.1 cm^2^ V^−1^ s^−1^) [13–17], high electron affinity, excellent self-assembling properties [18–20], and thermal and photochemical stabilities [21]. The excellent charge carrier mobility of PDIs has been explained by the intermolecular π–π interactions with an interplanar distance (3.3–3.5 Å) [22–25] that leads to the formation of large crystalline domains which influence charge transport. However, the microscale domains reduce the donor–acceptor interface which ultimately impacts on efficient exciton dissociation in OPV devices [12]. Therefore, it is important to further investigate other fused π systems to determine if this drawback can be overcome while maintaining the favourable properties of PDIs.

**Figure 1:**
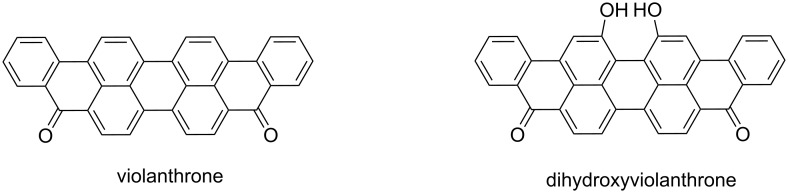
Chemical structures of violanthrone and dihydroxyviolanthrone.

Violanthrones are a class of materials featuring a large π-conjugated system composed of nine fused benzene rings with two carbonyl groups, in the 5 and 10 positions (Figure 1). The related structural features of violanthrones suggest that these materials may possess similar charge transport, optical and electrochemical properties to those of PDIs. However, the larger π-conjugated system of violanthrone, along with the two carbonyl groups, increases the possibility of stronger π–π intermolecular interactions which might result in a narrower HOMO–LUMO gap than that of PDI, and an absorption band extending to the near-infrared (NIR) region [26]. This makes violanthrone and its derivatives potential candidates for NIR optoelectronic applications. In fact, the intrinsic semiconducting properties of violanthrone is traced back to 1950, when Akamatu and Inokuchi measured its electrical conductivity (σ), which was found to be 3.4 × 10^−4^ Ω^−1^ cm^−1^ [27–28]. The chemical structure of violanthrone allows for its modification and hence the synthesis of materials with interesting spectral properties. Due to the low solubility of violanthrone in the majority of organic solvents, special attention has been drawn to its dihydroxy derivative (Figure 1), which allows further modification to the materials via etherification or esterification [29–30].

There has been a report on the structural modification of dihydroxyviolanthrone where the effect of three alkoxy substituents on the 16,17-*bis*(2-ethylhexyloxy)anthra[9,1,2-*cde*]benzo[*rst*]pentaphene-5,10-dione, on aggregation and photovoltaic properties was studied [30]. It was found that derivatives with the shortest linear alkyl chain (*n*-hexyl) exhibit the strongest π–π interactions since the distance between two adjacent molecules is shorter and less steric repulsion is observed. This was reflected by the highest hole mobility of the derivatives with *n*-hexyl chains (3.15 × 10^−4^ cm^2^ V^−1^ s^−1^), compared to derivatives with *n*-octyl chains (1.76 × 10^−4^ cm^2^ V^−1^ s^−1^) and 2-ethylhexyl chains (4.93 × 10^−5^ cm^2^ V^−1^ s^−1^). The stronger π–π interactions led to a higher power conversion efficiency (PCE) as a result of the higher short-circuit current density (*J*_sc_), due to films with higher crystallinity providing a smoother pathway to charge carriers to pass through the device [30].

**Figure 2:**
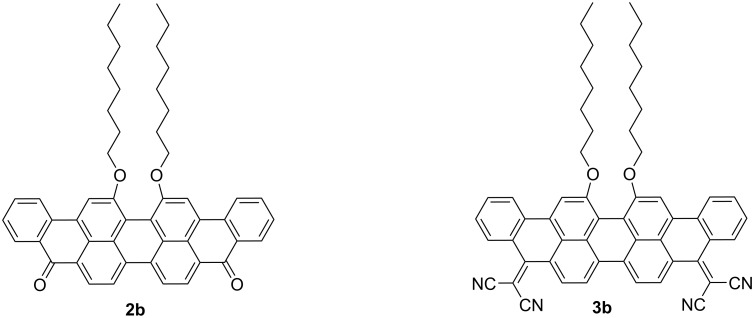
Chemical structures of **2b** and **3b**.

The π–π intermolecular interactions, the molecular stacking and mobility of a solution-processable violanthrone derivative has been studied. It was shown that π–π stacking can be enhanced in solution and in the solid state by adding a non-solvent (*n*-hexane) to chloroform. Therefore, the resulting film of the compound obtained from a solvent mixture of chloroform/*n*-hexane showed a hole mobility of an order of magnitude higher (4.44 × 10^−4^ cm^2^ V^−1^ s^−1^) than that of the film obtained from pure chloroform (4.93 × 10^−5^ cm^2^ V^−1^ s^−1^) [26]. Another study reported the capability of violanthrone **2b** to act as an electron acceptor in OPVs when blended with PDI as a co-acceptor, which showed an enhanced light harvesting and photocurrent generation compared to the device without violanthrone being incorporated (Figure 2) [31].

Liu et al. [32] reported a novel violanthrone derivative **3b** via the incorporation of the strong electron-withdrawing dicyanomethylene unit. The study suggested that **3b** could be a potential n-type material for OPVs. The incorporation of two dicyanomethylene groups resulted in a material with strong electron affinity and low reduction potential of −0.56 V vs NHE, and a λ_max_ at 701 nm with ε of 4.69 × 10^4^ L mol^−1^ cm^−1^ which might lead to the contribution of **3b** to the photocurrent.

All previous studies suggested that violanthrone and its derivatives display electronic functionality and could be potentially used in organic electronics. Nevertheless, to the best of our knowledge, neither OPV device fabrication nor the charge mobility of **3b** has been reported. Therefore, in this work, the synthesis of compound **3b** and other new analagous solution-processable derivatives are reported. The performance of these materials as the semiconductor layer in OFETs was studied to determine the effect of the different side chains.

## Results and Discussion

### Synthesis

**Scheme 1:**
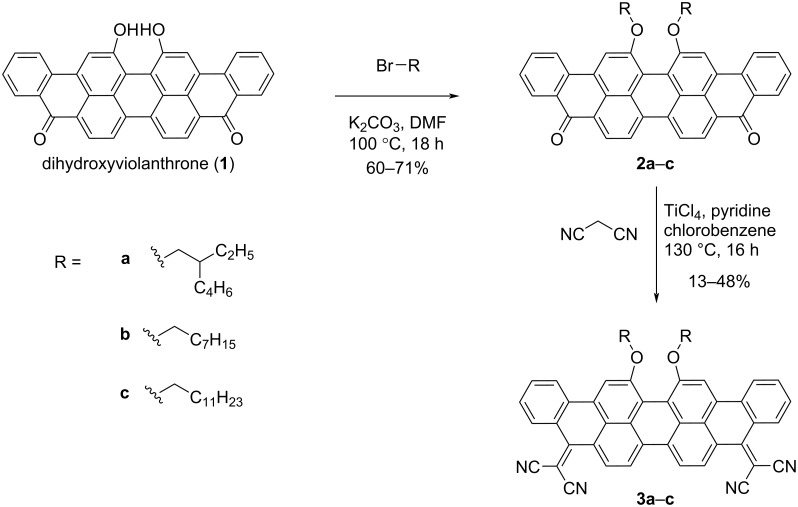
Synthesis of compounds **2a***–***c** and **3a***–***c**.

The synthesis of compounds **2a**–**c** and **3a**–**c** is shown in Scheme 1. Compounds **2a**–**c** were synthesised through a well-established etherification protocol [30] via the reaction of the commercially available compound 16,17-dihydroxyviolanthrone with 2-ethylhexyl bromide (**a**), 1-bromooctane (**b**), and 1-bromododecane (**c**) resulting in compounds, **2a**, **2b** and **2c**, respectively. The final target compounds **3a**–**c** were synthesised in 13%, 48% and 36% yield, respectively, following the reported procedure for anthraquinone, where the Knoevenagel condensation with malononitrile was successfully reported [33].

### Theoretical studies

Density functional theory (DFT) calculations of two derivatives of compounds **2** and **3**, having methoxy groups instead of the longer alkyl chains have been reported in the literature [32], providing information on the molecular structure and packing of these materials. However, no significant information about the electronic and orbital distributions was provided, therefore further investigation was needed, using a more suitable basis set. The molecules were geometrically optimised at the ground state using the B3LYP functional with the 6-311G (d,p) basis set. The geometry of the two molecules was found to be nearly identical to the ones previously reported, with the nine fused rings of compound **2** almost planar, compared to the more twisted geometry of compound **3**. This could possibly result in weak π–π interactions with the potential to form nanoscale pure and mixed domains in the bulk heterojunction on the length scale of the exciton diffusion length (5–15 nm), leading to an efficient exciton dissociation and charge generation [12].

**Figure 3:**
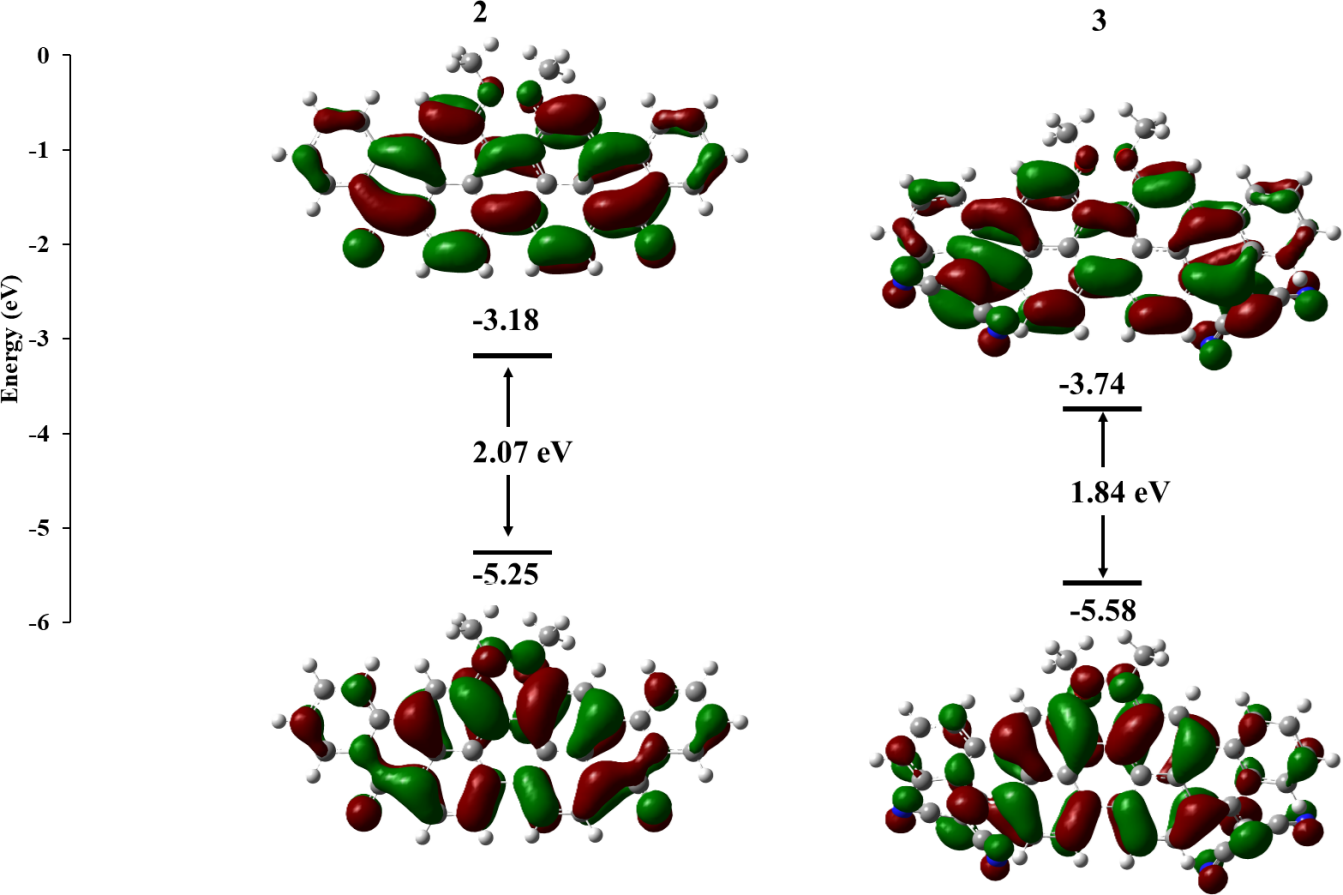
Optimised ground state geometries of compounds **2** and **3** calculated using B3LYP/6-311G(d,p) in the gas phase.

Figure 3 gives an insight into the spatial distribution and the energies of the frontier molecular orbitals of molecules **2** and **3**. In both cases, the highest occupied molecular orbital (HOMO) and the lowest unoccupied molecular orbital (LUMO) are uniformly delocalised throughout the nine fused rings, indicating that the two molecules could benefit from a potentially efficient and isotropic charge transport [12]. It is also evident that the presence of the two dicyanomethylene groups in compound **3** are responsible for lowering the energy of the two frontier molecular orbitals and for narrowing the energy gap between HOMO and LUMO. This is likely due to an enhanced push–pull effect in this molecule due to the presence of a stronger acceptor. Furthermore, the energy of the LUMO of compound **3** is comparable to reported PDI-based acceptors which have been used in OPVs with PCE > 7% [34].

### Crystallographic study

**Figure 4:**
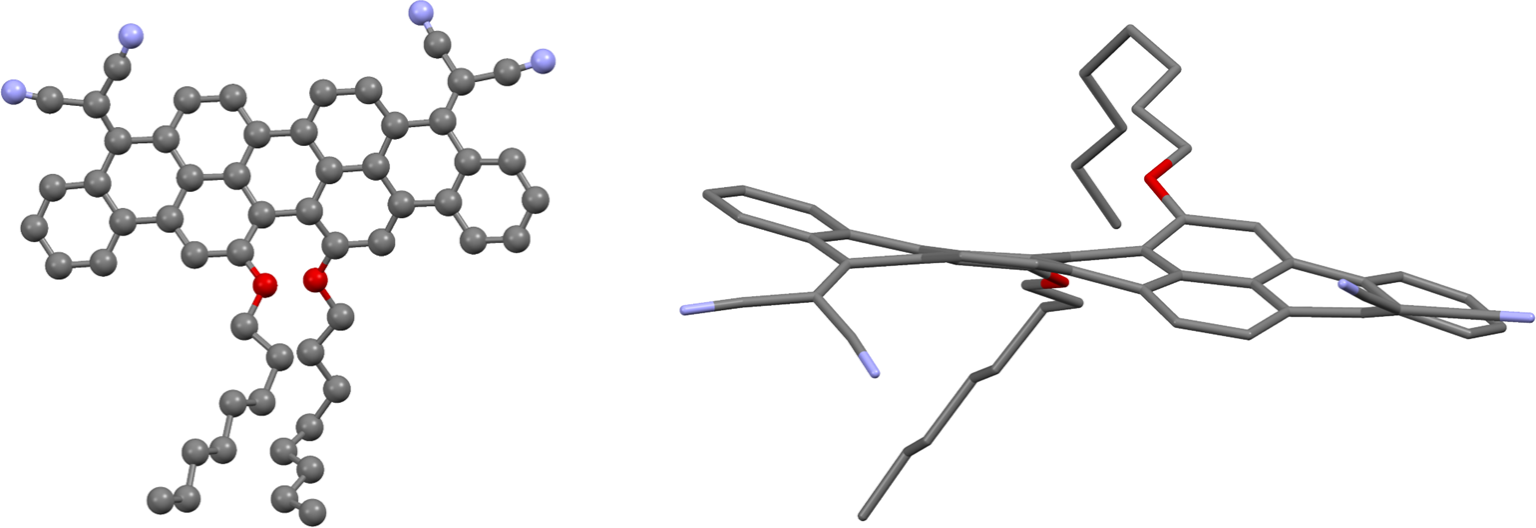
Views of the crystal structure of **3b** (left, shows displacement ellipsoids drawn at 50% probability level, right showing the twisted conformation).

Needle-shape crystals of compound **3b** suitable for single-crystal X-ray analysis were obtained by slow evaporation of a dichloromethane/isopropanol solution of **3b**. The crystal structure of **3b**, displayed in Figure 4, shows a very similar twisted conformation of the core of the molecule to that of the related methoxy-substituted structure obtained from theoretical studies [32] (Table S1 in Supporting Information File 1).

Molecules of **3b** form stacks along the *b*-axis linked by π–π interactions with centroid–centroid distances of 3.65 and 3.98 Å. These stacks lie in sheets with alternating aromatic–aliphatic layers (Figure S3 in Supporting Information File 1).

### Optical studies

**Figure 5:**
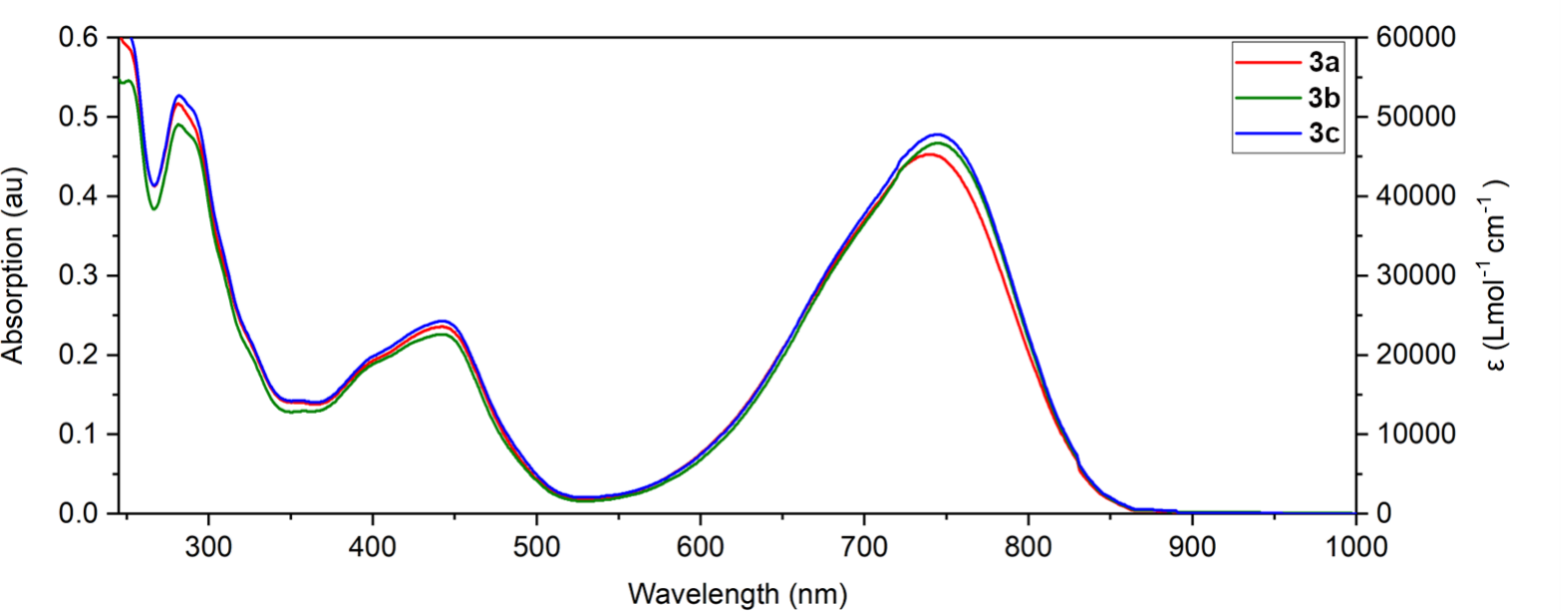
Absorption spectra of **3a**–**c** measured in dichloromethane solution (1 × 10^−5^ M).

The UV–vis absorption spectra of **3a**, **3b**, and **3c** are presented in Figure 5, and were carried out in dichloromethane solution (1 × 10^−5^ mol L^−1^). The absorption properties are summarised in Table 1. The UV–vis absorption spectra of the materials show a wide absorption band from 530 nm to 860 nm for all compounds. Compound **3a** shows a slight hypsochromic shift (λ_max_ = 741 nm) in comparison with **3b** (λ_max_ = 745 nm) and **3c** (λ_max_ = 746 nm). All compounds displayed very similar extinction coefficients between 45000 and 48000 L mol^−1^ cm^−1^. The optical gaps (*E*_opt_) were estimated from the onset values of absorption (λ_onset_), and little difference was found with values of 1.47 eV for **3b** and 1.46 eV for **3a** and **3c**. It is noted that the optical properties did not show a significant change upon altering the alkyl chains which indicates that different alkyl substituents have a minimal effect on the frontier orbitals.

**Table 1:** The optical and the electrochemical data of compounds **3a**–**c**.

	optical properties				electrochemical properties		

	λ_max_ (nm)	λ_onset_ (nm)	*E*_opt_ (eV)	ε (L mol^−1^ cm^−1^)	IE (eV)	EA (eV)	*E*_fund_ (eV)

**3a**	741	851	1.46	45300	−5.38	−4.11	1.27
**3b**	745	845	1.47	46700	−5.34	−4.09	1.25
**3c**	746	851	1.46	47800	−5.40	−4.15	1.25

### Electrochemical studies

**Figure 6:**
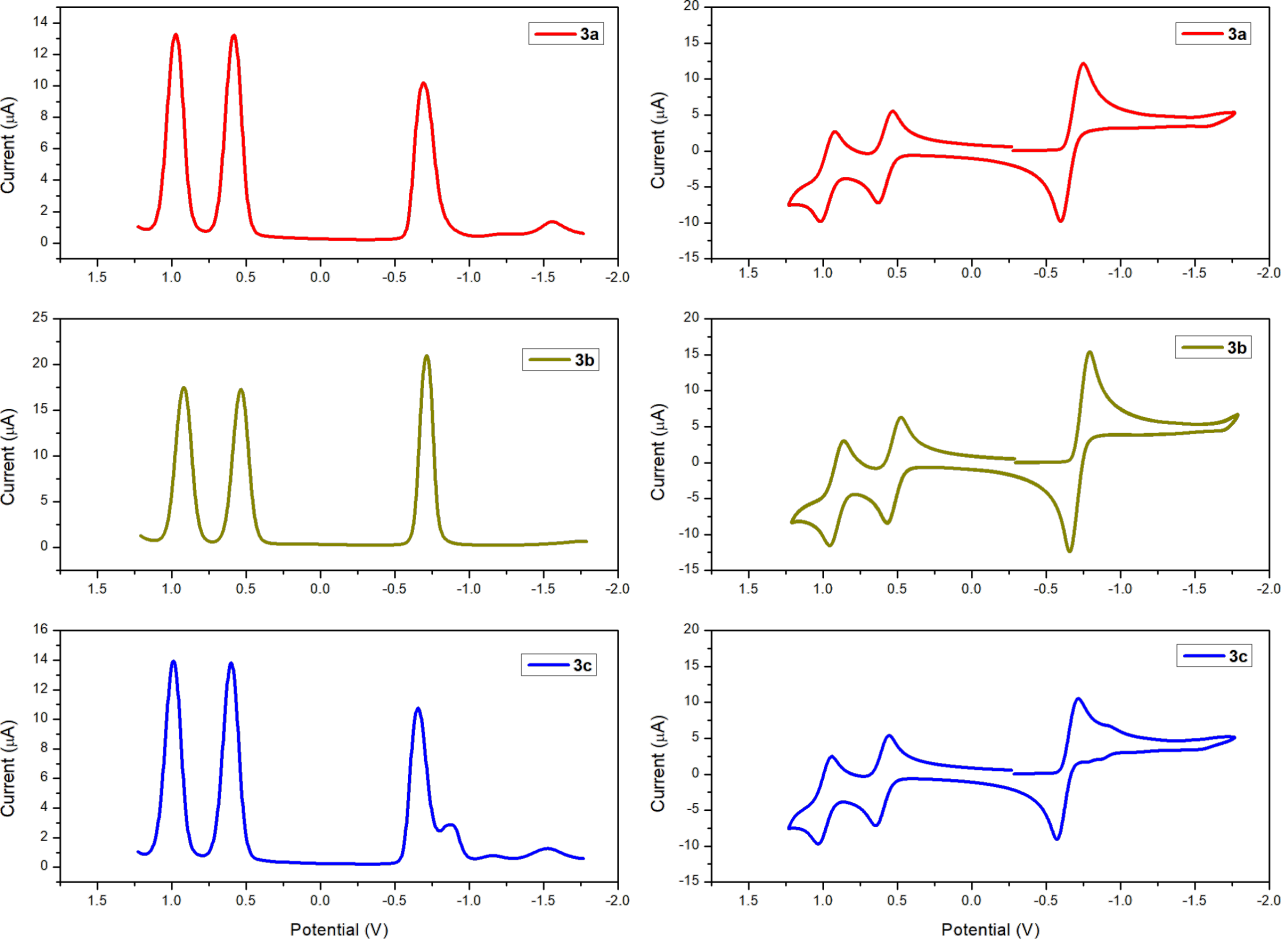
SWV (left) and CV (right) of compound **3a**–**c** (in dichloromethane 1 × 10^−3^ M) (V vs Fc/Fc^+^).

The electrochemical properties of the dyes were investigated by square wave voltammetry (SWV) and cyclic voltammetry (CV) and the data are summarised in Table 1, with the plots shown in Figure 6. All the materials exhibit at least one reversible reduction and two reversible oxidation waves as shown from the CV data (Figure 6 and Table 1). This reversibility is crucial for the regeneration of dyes following redox processes. The compounds display ionisation energies (IEs) of −5.38 eV, −5.34 eV, and −5.40 eV for **3a**, **3b**, and **3c**, respectively, with electron affinities (EAs) of −4.11 eV, −4.09 eV, and −4.15 eV. Consequently, the estimated *E*_fund_ are 1.27, 1.25 and 1.25 eV for **3a**, **3b**, and **3c**, respectively. The EA value is in a similar range (from −3.70 to −4.30 eV) of some of the most widely used fullerene-based acceptors such as PC_60_BM and PC_70_BM [35], which suggests that the three materials might function effectively as electron acceptors.

### OFET device studies

The electrical characteristics were confirmed by the fabrication of OFETs. Compounds **3a**–**c** were deposited by spin-coating onto n-doped Si/SiO_2_/Au substrates. The device performance parameters are summarised in Table 2, and their representative output and transfer curves are shown in Figure S5 (Supporting Information File 1). The charge mobility was determined in the saturation regime. The OFET devices based on **3a**–**c** showed only p-type charge transport, with the highest hole mobility obtained by **3b**. The hole mobilities (μ_h_) of **3b** and **3c** are calculated as 1.07 × 10^−2^ and 1.21 × 10^−3^ cm^2^ V^−1^s^−1^, respectively. However, the hole mobility of **3a** is only 3.62 × 10^−6^ cm^2^ V^−1^ s^−1^.

**Table 2:** Summary of OFET characteristics with **3a**–**c** materials.

	ON/OFF ratio	V_th_ (V)	μ_h_ (cm^2^ V^−1^ s^−1^)^a^

**3a**	10^2^	−3	3.62 × 10^−6^ (± 0.64 × 10^−7^)
**3b**	10^3^	−14	1.07 × 10^−2^ (± 0.44 × 10^−2^)
**3c**	10^4^	−19	1.21 × 10^−3^ (± 0.30 × 10^−3^)

^a^Calculated from an average of 8 devices. Standard deviation is listed in parentheses.

Side chain engineering is crucial for OFET performance since it plays an important role in determining solubility, molecular packing, polarity, and film-forming properties. Molecular packing, in particular, is greatly affected by alkyl chain length and branching point position [36]. Here, the difference in hole mobility among the three compounds can rationally be attributed to their distinguishing aggregate structures. Compared to **3a**, the linear side chains in compounds **3b** and **3c**, might favour intermolecular π–π interactions and crystallinity in the solid state, therefore providing an easier pathway for charge carriers to hop from one molecule to nearby molecules. The introduction of the bulky dicyanomethylene groups in **3b** resulted in a dramatic increase in μ_h_ (1.07 × 10^−2^ cm^2^ V^−1^s^−1^) compared to the previously reported **2b** (1.76 × 10^−4^ cm^2^ V^−1^s^−1^) [30]. On the other hand, the twisted configuration of the material’s backbone upon the introduction of dicyanomethylene groups, along with branched alkyl side chains in **3a**, might have contributed to a lower intermolecular π–π interaction and, therefore, lower μ_h_ (3.62 × 10^−6^ cm^2^ V^−1^ s^−1^) compared to the previously reported **2a** (4.93 × 10^−5^ cm^2^ V^−1^ s^−1^) [30].

## Conclusion

Violanthrone derivatives represent a promising group of semiconductor materials for organic electronics. It has been shown that molecular tailoring of violanthrone is simple and feasible. We have synthesised three soluble violanthrone derivatives with different side chains and found that due to the introduction of the electron-deficient dicyanomethylene groups, along with the extended π-conjugated framework, all compounds exhibit a narrow HOMO–LUMO gap (1.46–1.47 eV), with a wide absorption range exceeding 800 nm compared to their previously reported precursors [30]. The electrochemical studies of the three materials show reversible oxidation and reduction waves with EA values that are in a similar range (from −3.70 to −4.30 eV) of some of the most widely used fullerene-based acceptors such as PC_60_BM and PC_70_BM [35], which suggests that the three materials might function well as components in OPVs. Among the three materials the introduction of dicyanomethylene groups to compound **2b** significantly improved the μ_h_ by 60-fold. It is also notable that **3a** bearing branched 2-ethylhexyl side chains showed inferior performance compared to the isomeric **3b** with linear *n*-octyl chains. The poor device performance is most likely caused by branched side chains that might induce a stronger disorder in the film, which results in hindered charge transport.

## Experimental

### Computational

Density functional theory (DFT) calculations were performed using Gaussian 09 software. Molecular geometries were initially optimised semi-empirically (AM1) and then reoptimised by DFT using the B3LYP method with the 6-311G(d,p) basis set unless stated otherwise. The absence of transition states was confirmed by the absence of imaginary frequencies in vibrational frequency calculations. The long side chains were replaced by methyl units to aid the convergence of the geometry optimisations.

### Crystallography

Single crystal X-ray diffraction data for **3b** were collected by the EPSRC National Crystallography Service using a ROD, Synergy Custom system, HyPix diffractometer with Cu Kα radiation, λ = 1.54178 Å. Data were collected and processed using CrysAlis PRO 1.171.39.30d (Rigaku OD, 2015). The structure was solved using SHELXT 2018/2 [37] and refined using SHELXL 2018/3 [38] within Olex2 1.3 [39]. Non-H atoms were refined with anisotropic atomic displacement parameters (ADPs) and H-atoms were placed in geometrically calculated positions and included as part of a riding model except the Me H-atoms which were included as a rigid rotor.

### Organic field-effect transistors (OFETs) fabrication and measurement

Bottom-gate, bottom-contact organic field-effect transistors were made using prefabricated substrates (Fraunhofer IPMS, product code 1301). The substrates consisted of an n-doped Si gate electrode, SiO_2_ (230 nm) dielectric layer and Au (30 nm + 10 nm ITO adhesion layer) interdigitated source and drain electrodes, 1 cm in width. The substrate contained source-drain electrodes at channel lengths of 20, 10, 5, and 2.5 μm. For all compounds, four devices of 20 μm channel length and four devices with 10 μm channel length were tested, with the exception of **2b** where some devices failed due to high resistance and testing was carried out using 5 μm channel length due to the low currents measured at higher channel lengths. Testing was carried out using a Keithley 4200 Semiconductor Characterisation System. Charge mobility was calculated in the saturation regime.

The substrates were washed using deionised H_2_O, acetone, and isopropanol before being dried over a stream of compressed air. Octadecyltrichlorosilane (30 μM) was dropcast onto the substrate for 5 minutes before the substrate was washed with toluene. The substrate was then dried over compressed air. A solution (10 mg mL^−1^ in CHCl_3_) of the semiconductor material was deposited by spin-coating at 1000 rpm for 60 seconds.

### Synthesis

#### Compound **2a**

16,17-Dihydroxyviolanthrone (500 mg, 1.02 mmol) and 2-ethylhexyl bromide (550 μL, 3.06 mmol) were dissolved in *N*,*N*-dimethylformamide (30 mL). Then, potassium carbonate was added (300 mg, 2.04 mmol), and the reaction mixture was stirred at 100 °C overnight. After cooling the reaction mixture to room temperature, it was poured into methanol (200 mL), and the resulting precipitate was filtered, then washed with water (150 mL) to give the title compound as a dark solid (440 mg, 60%). ^1^H NMR (400 MHz, CDCl_3_) δ 8.79 (d, *J* = 8.0 Hz, 2H), 8.65 (d, *J* = 8.1 Hz, 2H), 8.56 (d, *J* = 7.6 Hz, 2H), 8.40 (d, *J* = 7.8 Hz, 2H), 8.30 (s, 2H), 7.82 (t, *J* = 7.6 Hz, 2H), 7.62 (t, *J* = 7.4 Hz, 2H), 4.05 (m, 4H), 1.77 (m, 2H), 1.38 (m, 16H), 0.93–0.51 (m, 12H); ^13^C NMR (100 MHz, CDCl_3_) δ 183.4, 157.1, 135.7, 134.7, 133.3, 131.1, 129.6, 128.6, 128.3, 127.9, 127.7, 127.3, 123.9, 123.3, 122.9, 117.6, 114.6, 65.5, 42.1, 40.1, 30.2, 29.2, 23.5, 23.2, 14.2, 11.2; ASAP–HRMS (*m*/*z*): [M + H]^+^ calcd for C_50_H_49_O_4_, 713.3646; found 713.3631.

#### Compound **2b**

16,17-Dihydroxyviolanthrone (2.00 g, 4.09 mmol) and 1-bromooctane (2.12 mL, 12.28 mmol) were dissolved in *N*,*N*-dimethylformamide (60 mL). Then, potassium carbonate was added (1.13 g, 8.19 mmol), and the reaction mixture was stirred at 100 °C overnight. After cooling the reaction mixture to room temperature, it was poured into methanol (400 mL), and the resulting precipitate was filtered, then washed with water (300 mL) to give the title compound as a dark solid (1.90 g, 65%). ^1^H NMR (400 MHz, CDCl_3_) δ 8.72 (d, *J* = 8.0 Hz, 2H), 8.60–8.47 (m, 4H), 8.37 (d, *J* = 8.2 Hz, 2H), 8.27 (s, 2H), 7.80 (t, *J* = 7.2 Hz, 2H), 7.60 (t, *J* = 7.3 Hz, 2H), 4.25 (br, 4H), 1.94–1.80 (m, 4H), 1.34 (d, *J* = 90.2 Hz, 20H), 0.82 (d, *J* = 6.9 Hz, 6H); ^13^C NMR (100 MHz, CDCl_3_) δ 183.2, 156.3, 135.6, 134.5, 133.2, 131.0, 129.4, 128.6, 128.3, 127.7, 127.5, 127.1, 123.6, 123.2, 122.7, 117.2, 113.5, 69.8, 31.9, 29.9, 29.6, 29.5, 26.2, 22.8, 14.2; HRESIMS (*m*/*z*): [M + Na]^+^ calcd for C_50_H_48_NaO_4_, 753.3409; found, 735.3445.

#### Compound **2c**

16,17-Dihydroxyviolanthrone (500 mg, 1.02 mmol) and 1-bromododecane (800 μL, 3.06 mmol) were dissolved in *N*,*N*-dimethylformamide (30 mL). Then, potassium carbonate was added (300 mg, 2.04 mmol), and the reaction mixture was stirred at 100 °C overnight. After cooling the reaction mixture to room temperature, it was poured into methanol (200 mL), and the resulting precipitate was filtered, then washed with water (150 mL) to give the title compound as a dark solid (600 mg, 71%). ^1^H NMR (400 MHz, CDCl_3_) δ 8.78 (d, *J* = 8.0 Hz, 2H), 8.63 (d, *J* = 8.3 Hz, 2H), 8.57 (d, *J* = 7.7 Hz, 2H), 8.39 (d, *J* = 8.0 Hz, 2H), 8.30 (s, 2H), 7.81 (t, *J* = 7.4 Hz, 2H), 7.62 (t, *J* = 7.6 Hz, 2H), 4.26 (s, 4H), 1.92–1.72 (m, 4H), 1.55–1.02 (m, 36H), 0.86 (t, *J* = 6.8 Hz, 6H); ^13^C NMR (100 MHz, CDCl_3_) δ 183.2, 156.4, 135.6, 134.5, 133.2, 131.1, 129.5, 128.6, 128.3, 127.8, 127.5, 127.2, 123.7, 123.2, 122.8, 117.3, 113.6, 69.8, 63.2, 32.9, 32.0, 29.7, 29.6, 29.5, 29.4, 26.2, 25.8 , 22.8, 14.2; ASAP–HRMS (*m*/*z*): [M + H]^+^ calcd for C_58_H_64_O_4_, 825.4875; found, 825.4883.

#### Compound **3a**

Compound **2a** (200 mg, 0.280 mmol) and malononitrile (100 mg, 0.840 mmol) were dissolved in anhydrous chlorobenzene (6 mL). To the dark blue mixture titanium tetrachloride (100 μL, 0.840 mmol) and pyridine (130 μL, 1.68 mmol) were added and the mixture was stirred under reflux overnight. After cooling the reaction mixture to room temperature, it was poured into ice-water (50 mL) and extracted with dichloromethane (3 × 20 mL). The combined organic extract was dried over MgSO_4_, filtered and concentrated under reduced pressure. The crude product was purified by silica column chromatography (SiO_2_, CH_2_Cl_2_/diethyl ether 98:2) to give the title compound as a dark solid (30.0 mg, 13%). Mp 295–296 °C; ^1^H NMR (400 MHz, CDCl_3_) δ 8.75 (d, *J* = 8.2 Hz, 2H), 8.55 (dd, *J* = 12.5, 8.2 Hz, 4H), 8.35 (d, *J* = 8.1 Hz, 2H), 8.25 (s, 2H), 7.80 (t, *J* = 7.4 Hz, 2H,), 7.63 (t, *J* = 7.7 Hz, 2H), 4.24–3.96 (m, 4H), 1.79 (br, 2H), 1.54–1.21 (m, 16H), 0.84 (m, 12H); ^13^C NMR (100 MHz, CDCl_3_) δ 183.4, 157.1, 135.7, 134.7, 133.3, 131.1, 129.6, 128.6, 128.3, 127.9, 127.7, 127.3, 123.9, 123.3, 122.9, 117.6, 114.6, 65.5, 42.1, 40.1, 30.2, 29.2, 23.2 (C, 14.2, 11.2; ASAP–HRMS (*m*/*z*): [M + H]^+^ calcd for C_56_H_49_N_4_O_2_, 809.3841; found, 809.3856 .

#### Compound **3b**

Compound **2b** (200 mg, 0.28 mmol) and malononitrile (60.0 mg, 0.840 mmol) were dissolved in anhydrous chlorobenzene (6 mL). To the dark blue mixture, titanium tetrachloride (50.0 μL, 0.420 mmol) and pyridine (70.0 μL, 0.84 mmol) were added and the mixture was stirred under reflux overnight. After cooling the reaction mixture to room temperature, it was poured into ice-water (50 mL) and extracted with dichloromethane (3 × 20 mL). The combined organic extract was dried over MgSO_4_, filtered and concentrated under reduced pressure. The crude product was purified by column chromatography (SiO_2_, petroleum ether/CH_2_Cl_2_ 1:9) to give the title compound as a dark solid (110 mg, 48%). Analysis is in agreement with previously reported data [32]. Mp 294–295 °C; ^1^H NMR (400 MHz, CDCl_3_) δ 8.75 (d, *J* = 8.2 Hz, 2H), 8.56 (dd, *J* = 8.1, 1.0 Hz, 2H), 8.51 (d, *J* = 8.4 Hz, 2H), 8.35 (d, *J* = 7.9 Hz, 2H), 8.26 (s, 2H), 7.82–7.75 (m, 2H), 7.65–7.58 (m, 2H), 4.31 (s, 4H), 1.94–1.84 (m, 4H), 1.54–1.07 (m, 20H), 0.89–0.78 (m, 6H); ^13^C NMR (100 MHz, CDCl_3_) δ 161.0, 157.1, 133.8, 132.9, 132.6, 129.1, 129.0, 128.5, 128.3, 127.9, 127.2, 127.1, 124.2, 122.5, 120.9, 117.6, 116.3, 112.5, 76.8, 70.0, 31.9, 29.8, 29.5, 29.4, 26.1, 22.7, 14.2; FAB^+^–HRMS (*m*/*z*): [M + H]^+^ calcd for C_56_H_49_N_4_O_2_, 809.3856; found, 809.3879 .

Details of the crystal structure of **3b** are given in the CIF which can be obtained from the CCDC free of charge CCDC 2128169 from the Cambridge Crystallographic Data Centre [40].

#### Compound **3c**

Compound **2c** (300 mg, 0.360 mmol) and malononitrile (100 mg, 1.08 mmol) were dissolved in anhydrous chlorobenzene (6 mL). To the dark blue mixture titanium tetrachloride (120 μL, 1.08 mmol) and pyridine (170 μL, 2.16 mmol) were added and the mixture was stirred under reflux overnight. After cooling the reaction mixture to room temperature, it was poured into ice-water (50 mL) and extracted with dichloromethane (3 × 20 mL). The combined organic extracts were dried over MgSO_4_, filtered and concentrated under reduced pressure. The crude product was purified by silica column chromatography (SiO_2_, CH_2_Cl_2_/diethyl ether 98:2) to give the title compound as a dark solid (120 mg, 36%). Mp 241–242 °C; ^1^H NMR (400 MHz, CDCl_3_) δ 8.60 (d, *J* = 8.2 Hz, 2H), 8.48 (d, *J* = 8.1 Hz, 2H), 8.35 (d, *J* = 8.0 Hz, 2H), 8.30 (d, *J* = 8.3 Hz, 2H), 8.24 (s, 2H), 7.78 (t, *J* = 7.3 Hz, 2H), 7.57 (t, *J* = 7.7 Hz, 2H), 4.34 (s, 4H), 1.98–1.86 (m, 4H), 1.49–1.18 (m, 36H), 0.87 (t, *J* = 6.9 Hz, 6H); ^13^C NMR (100 MHz, CDCl_3_) δ 183.2, 156.4, 135.6, 134.5, 133.2, 131.1, 129.5, 128.6, 128.3, 127.8, 127.5, 127.2, 123.7, 123.2, 122.8, 117.3, 113.6, 69.8, 63.2, 32.9, 32.0, 29.9, 29.7, 29.5, 29.4, 26.2, 25.8, 22.8, 14.2; ASAP–HRMS (*m*/*z*): [M + H]^+^ calcd for C_64_H_65_N_4_O_2_, 921.5107; found, 921.5108.

## Supporting Information

File 1 NMR spectra of compounds, crystallographic informnation and OFET plots.

## Data Availability

Data generated and analysed during this study is available from the corresponding author upon reasonable request.
