# Supplementary material for: The charge transport properties of dicyanomethylene-functionalised violanthrone derivatives
Source: Beilstein J Org Chem. 2024 Nov 13;20:2921–30. doi: 10.3762/bjoc.20.244 (PMC11572013; doi:10.3762/bjoc.20.244)
Supplement: File 1 — NMR spectra of compounds, crystallographic informnation and OFET plots. [file Beilstein_J_Org_Chem-20-2921-s001.pdf]

BEILSTEIN JOURNAL OF ORGANIC CHEMISTRY

Supporting Information

for

The charge transport properties of dicyanomethylene-functionalised violanthrone derivatives

Sondos A. J. Almahmoud, Joseph Cameron, Dylan Wilkinson, Michele Cariello, Claire Wilson, Alan A. Wiles, Peter J. Skabara and Graeme Cooke

Beilstein J. Org. Chem. **2024,** *20,* 2921–2930. doi:10.3762/bjoc.20.244

NMR spectra of compounds, crystallographic informnation and OFET plots

License and Terms: This is a supporting information file under the terms of the Creative Commons Attribution License (https://creativecommons.org/licenses/by/4.0). Please note that the reuse, redistribution and reproduction in particular requires that the author(s) and source are credited and that individual graphics may be subject to special legal provisions.
The license is subject to the *Beilstein Journal of Organic Chemistry* terms and conditions: (https://www.beilstein-journals.org/bjoc/terms)

	Table of contents	Page
**1 Materials**		S2
**2 NMR Spectra**		S3–S8
	^{1}H NMR of compound **2a**	S3
	^{13}C NMR of compound **2a**	S3
	^{1}H NMR of compound **2b**	S4
	^{13}C NMR of compound **2b**	S4
	^{1}H NMR of compound **2c**	S5
	^{13}C NMR of compound **2c**	S5
	^{1}H NMR of compound **3a**	S6
	^{13}C NMR of compound **3a**	S6
	^{1}H NMR of compound **3b**	S7
	^{13}C NMR of compound **3b**	S7
	^{1}H NMR of compound **3c**	S8
	^{13}C NMR of compound **3c**	S8
**3 Crystallographic studies**		S9–S23
Figure S1	View of the crystal structure of **3b**, displacement ellipsoids drawn at 50% probability level, H-atoms omitted for clarity. Selected atom labels given for torsion angles in Table S1.	S9
Table S1	Selected torsion angles for **3b** and comparison with calculated values for Va-CN (OMe substituent) from Zhu et al.[1]	S10
Figure S2	Packing diagram of the structure of **3b**, viewed along the *b*-axis, showing the stacks of molecules linked by π–π interactions and the alternating aromatic and aliphatic layers.	S10
Figure S3	View showing π–π stacking in **3b**; two distinct π–π interactions between parallel rings with centroid-centroid distances of 3.65 and 3.98 Å	S11
**4 OFET Plots**		S24–S25
Figure S4:	(a) Output and (b) transfer characteristics (V_{DS} = −70 V) of OFET fabricated using **3b** layer. Channel width = 1 cm, channel length = 20 μm	S24
Figure S5:	(a) Output and (b) transfer characteristics (V_{DS} = −70 V) of OFET fabricated using **2b** layer. Channel width = 1 cm, channel length = 10 μm.	S24
Figure S6:	(a) Output and (b) transfer characteristics (V_{DS} = −70 V) of OFET fabricated using **2a** layer. Channel width = 1 cm, channel length = 20 μm	S24
Figure S7:	(a) Output and (b) transfer characteristics (V_{DS} = −70 V) of OFET fabricated using **3c** layer. Channel width = 1 cm, channel length = 20 μm	S25
Figure S8:	(a) Output and (b) transfer characteristics (V_{DS} = −70 V) of OFET fabricated using **3a** layer. Channel width = 1 cm, channel length = 20 μm	S25

[1] B. Liu, D. Fan, Q. Zhang, Y. Chen and W. Zhu, Frontiers of Chemistry in China, 2010, 5, 200-207.

1 Materials and characterisation procedures

All reagents were purchased from either Acros®, Alfa Aesar®, Fisher Scientific® Fluorochem®, Sigma Aldrich®, and TCI®. Column chromatography was carried out using silica gel 40–63 nm 60 Å (Sigma-Aldrich). TLCs were performed using Merck silica gel 60 covered aluminium plates F254. Dry solvents were obtained from either solvent purification system (activated alumina columns) (Pure Solv 400-5-MD) or Sigma Aldrich®. ^{1}H NMR and ^{13}C NMR spectra were recorded on a Bruker AVIII (400 MHz) spectrometer, operating at 400 MHz and 100 MHz, respectively. Mass spectra were obtained from either the mass spectrometry service at the University of Glasgow or the EPSRC UK National Mass Spectrometry Facility at Swansea University. UV–vis measurements were carried out using a Perkin Elmer Lambda 25 spectrometer and Shimadzu UV-3600 UV–vis–NIR spectrophotometer. Square wave voltammetry was recorded at room temperature under nitrogen on a CH instruments Electrochemical Workstation 440A using a three-electrode cell with a platinum (Pt) working electrode, a Pt wire counter electrode and an Ag wire pseudo reference electrode. Samples were analysed at 1.0 mM concentrations with a scan rate of 0.1 V s^{-1} using $TBAPF_6$ (0.1 M in corresponding solvent) as the supporting electrolyte. The redox potentials are referenced to ferrocene (internal or external reference) with the Fc/Fc^{+} redox couple adjusted to 0.0 V. For the estimations of energy levels of the analytes, the HOMO energy of ferrocene was taken as 4.80 eV. All the spectroscopic and electrochemical data were processed using Origin Pro 8.5 software suite. The single crystal diffraction data were collected by the EPSRC National Crystallography Service. Details are given in the CIF which can be obtained from the CCDC free of charge CCDC 2128169.

2 NMR Spectra

^{1}H NMR of compound 2a

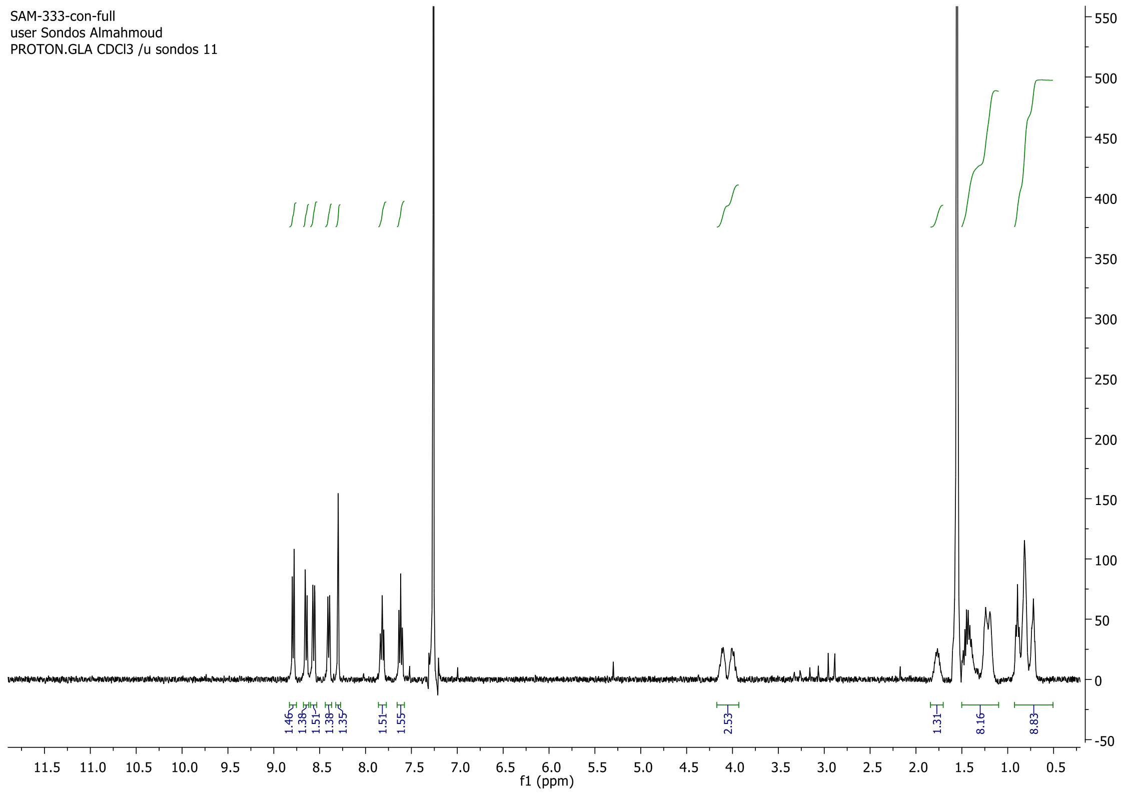

^{13}C NMR of compound 2a

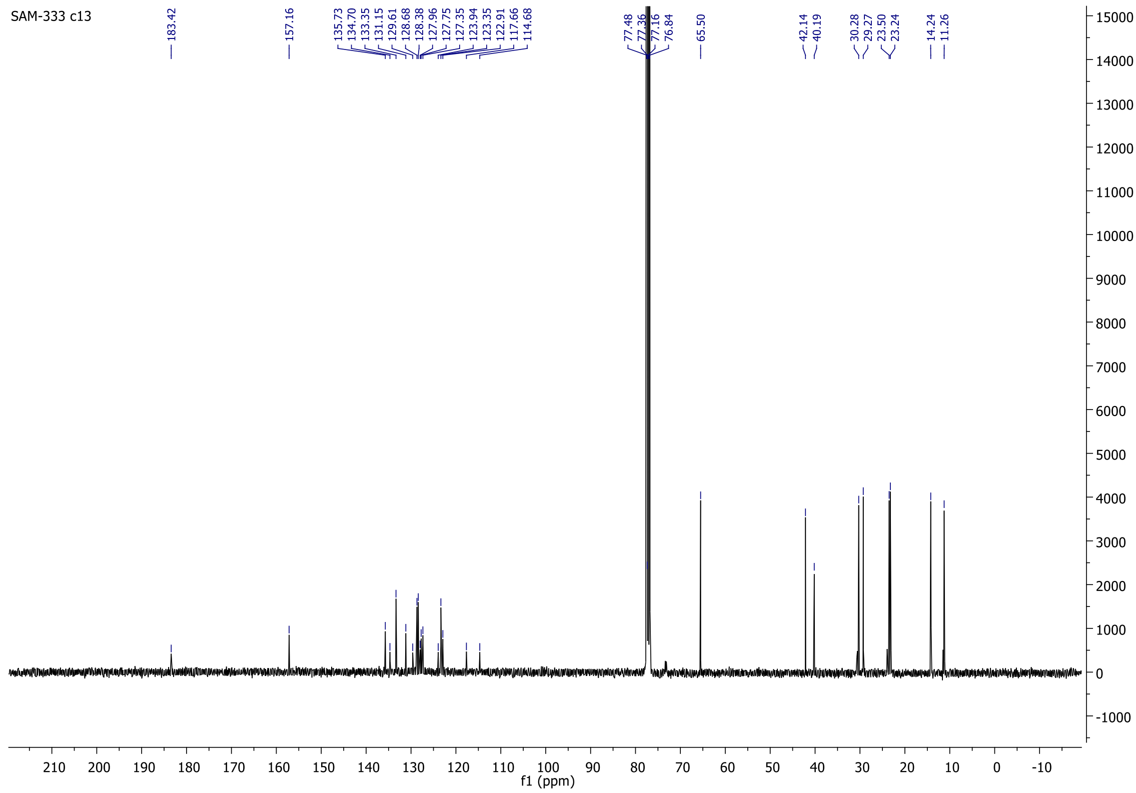

^{1}H NMR of compound 2b

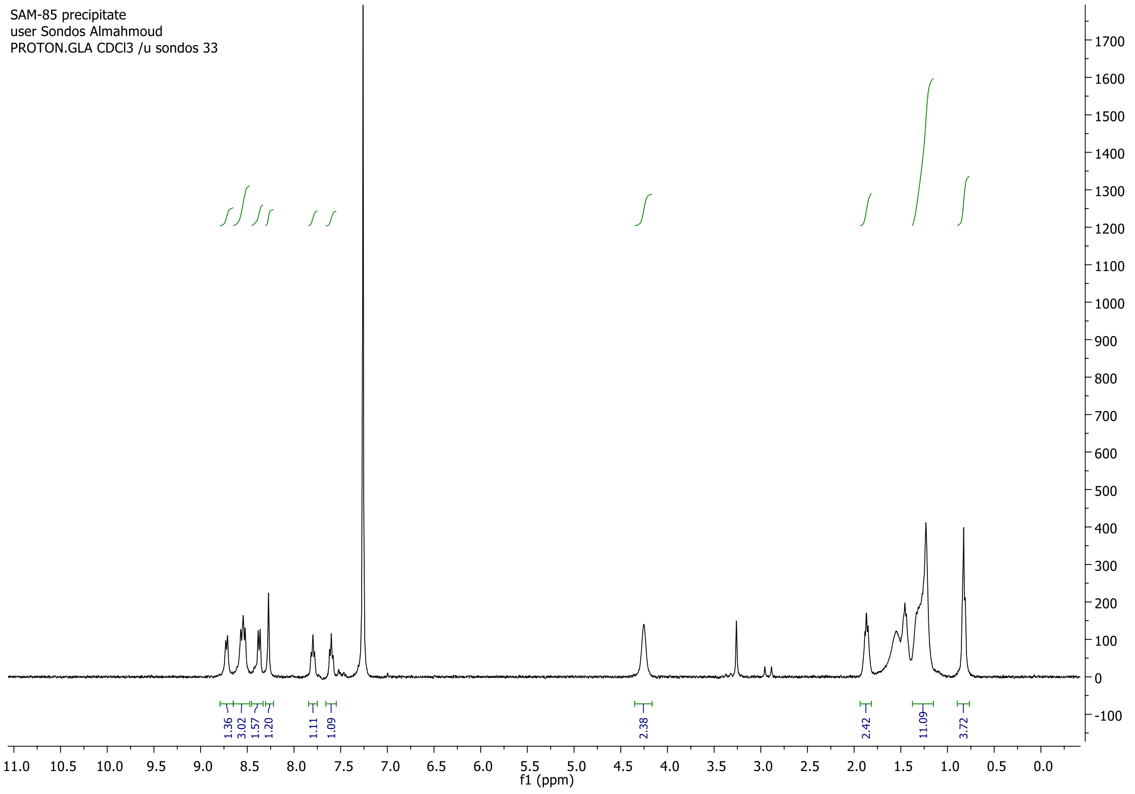

^{13}C NMR of compound 2b

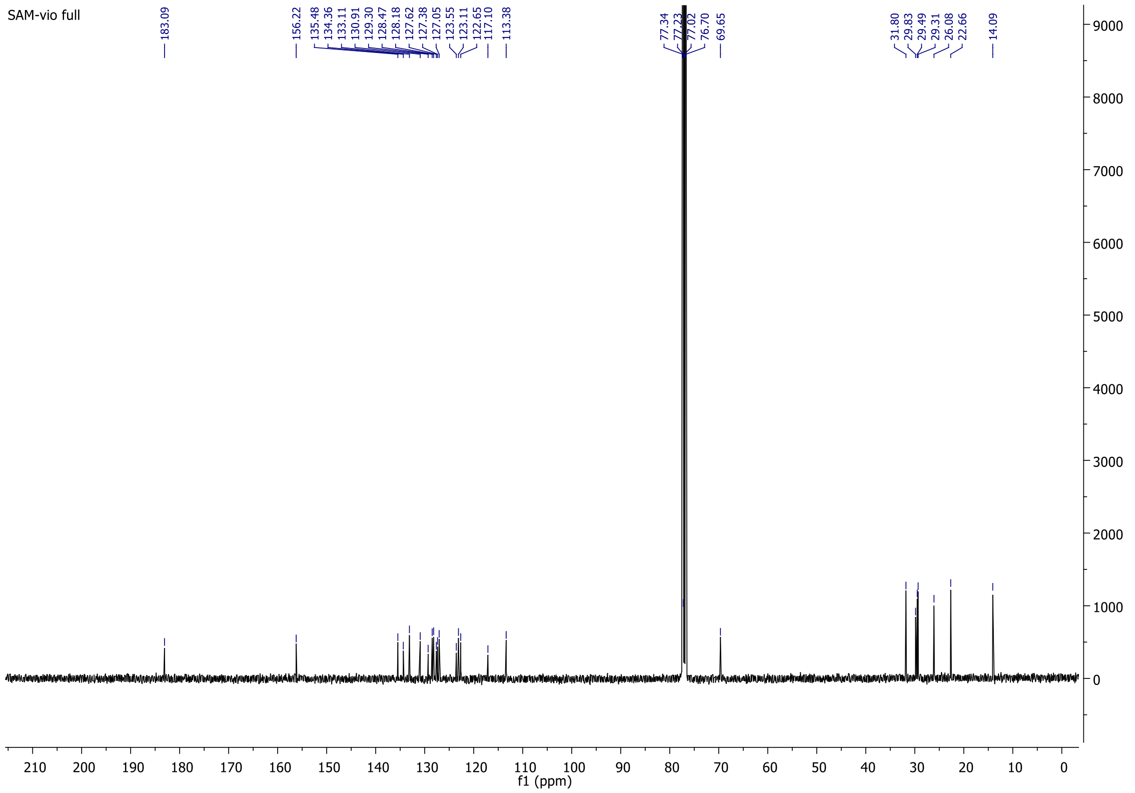

^{1}H NMR of compound 2c

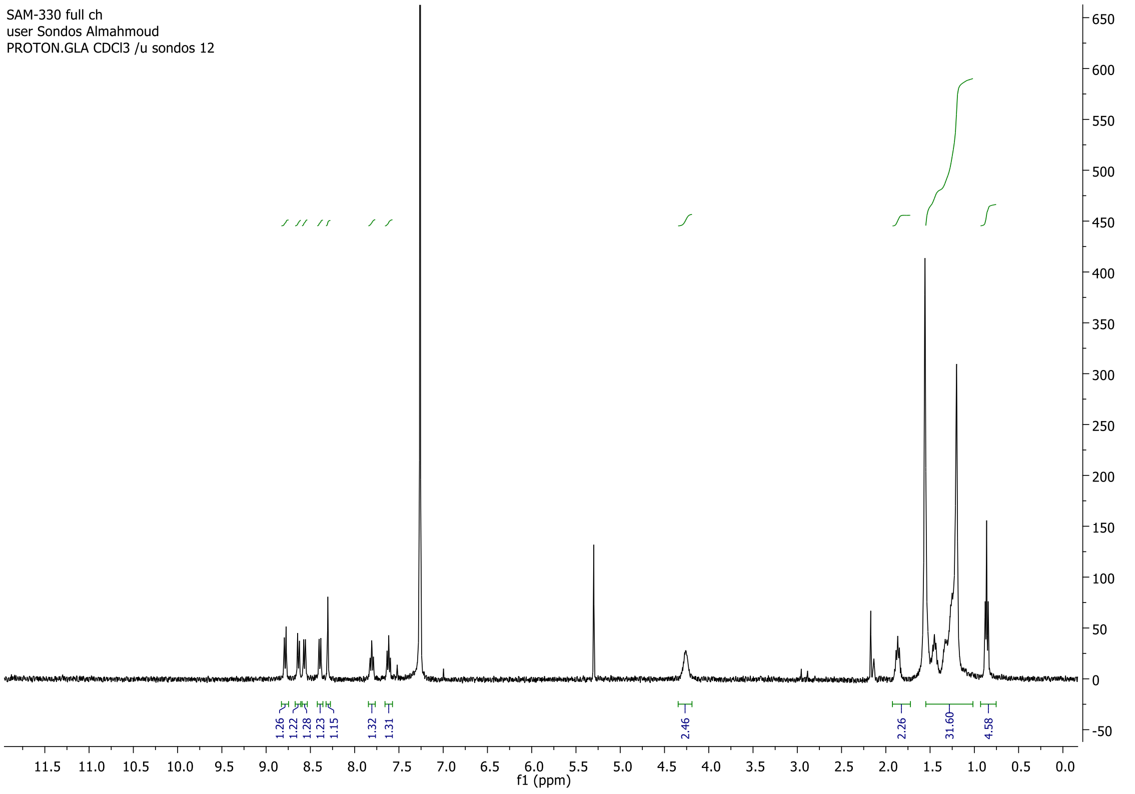

^{13}C NMR of compound 2c

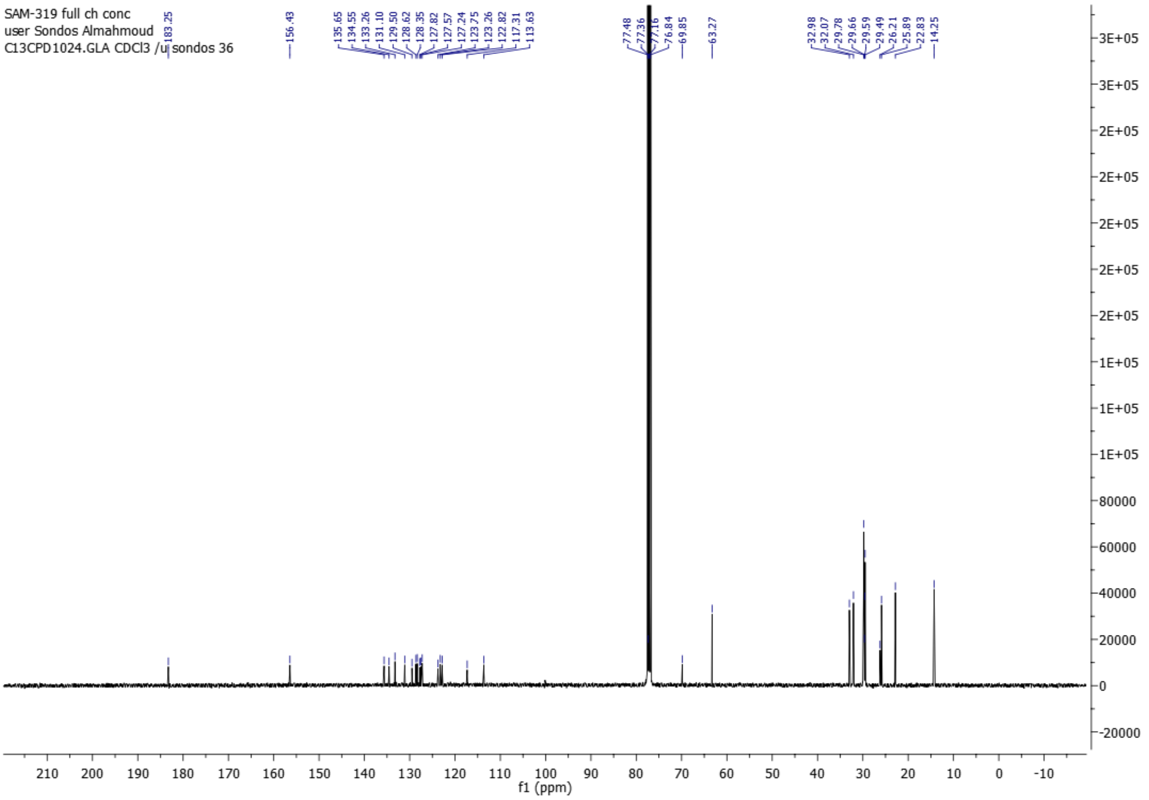

^{1}H NMR of compound 3a

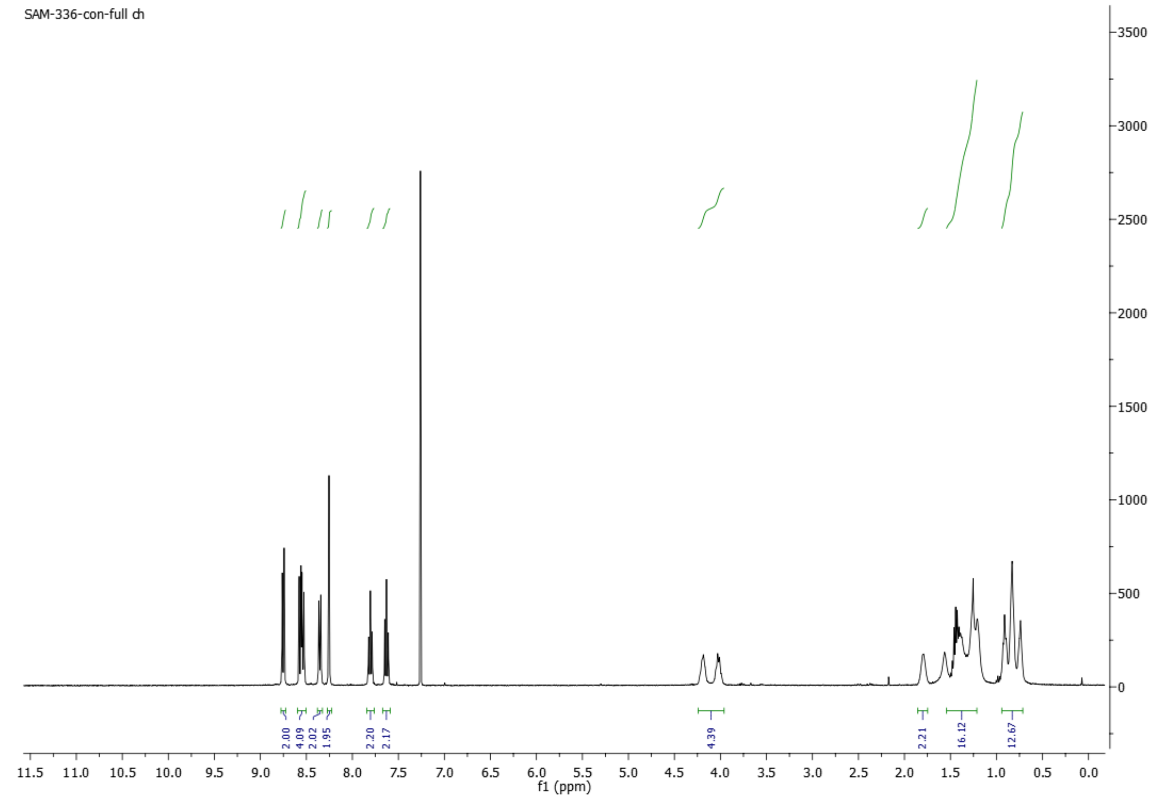

^{13}CNMR of compound 3a

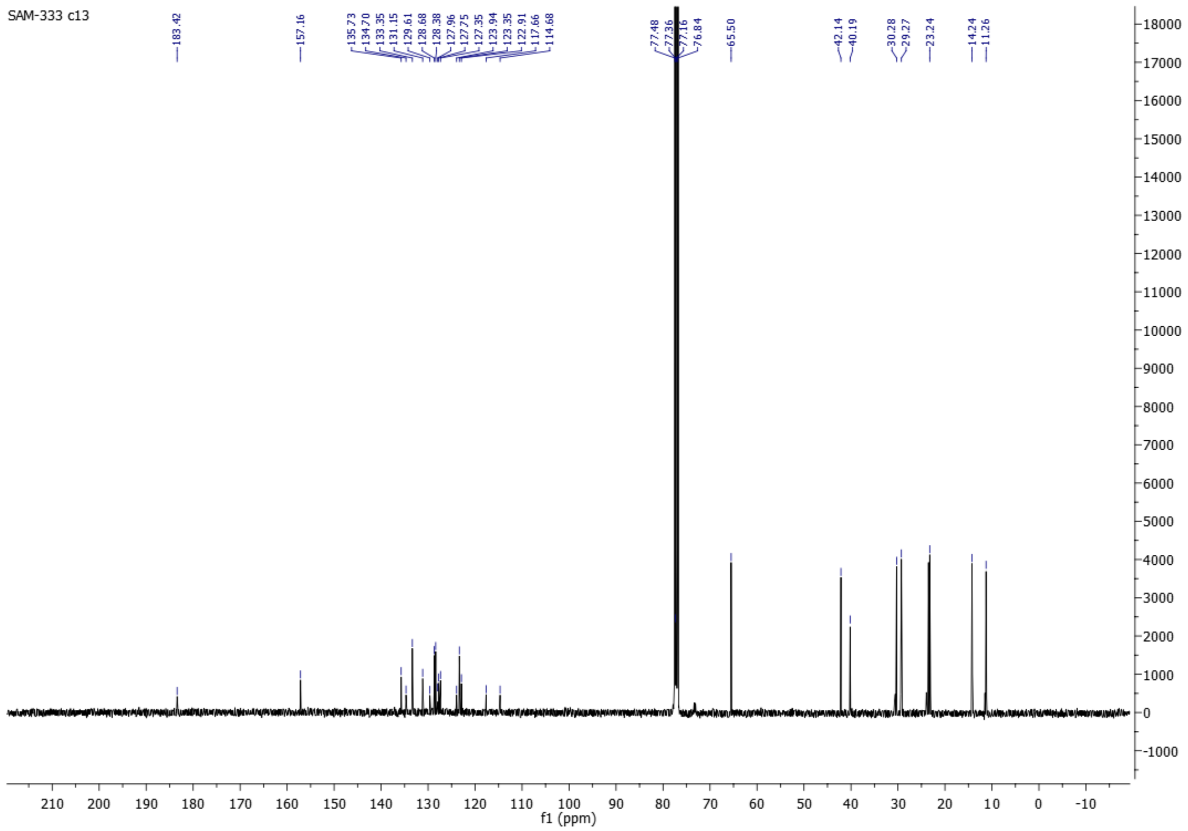

^{1}H NMR of compound 3b

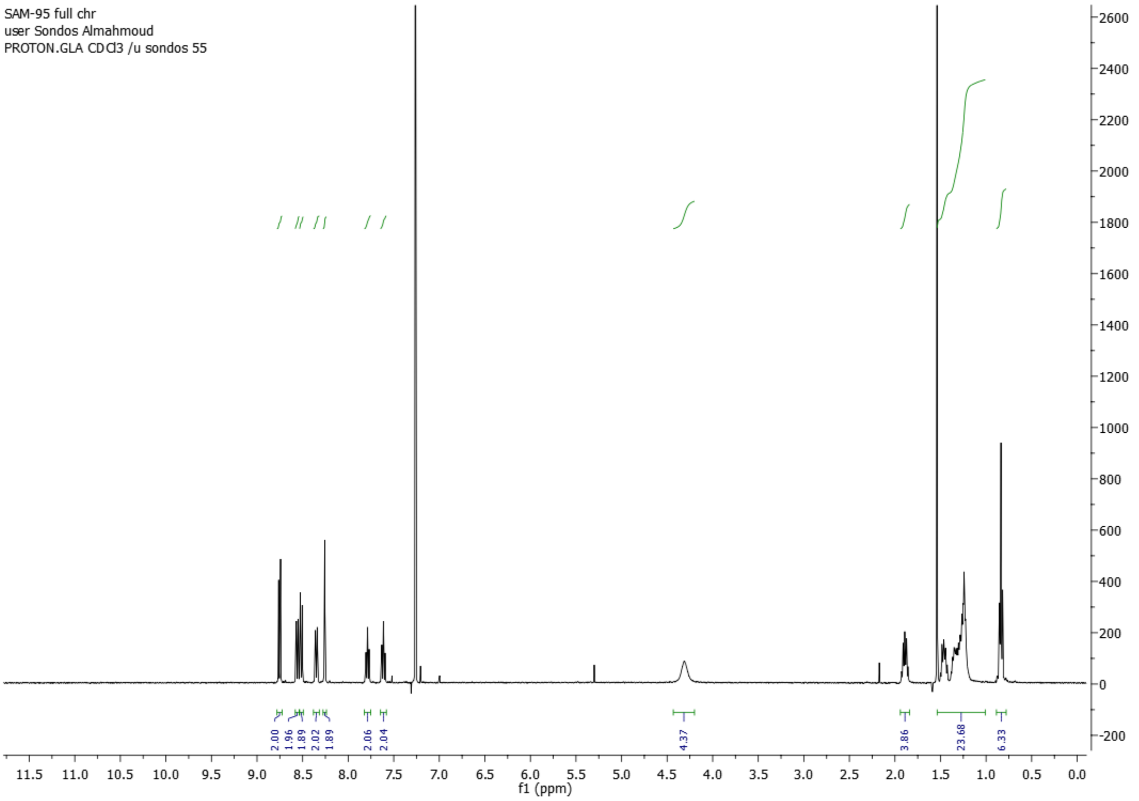

^{13}C NMR of compound 3b

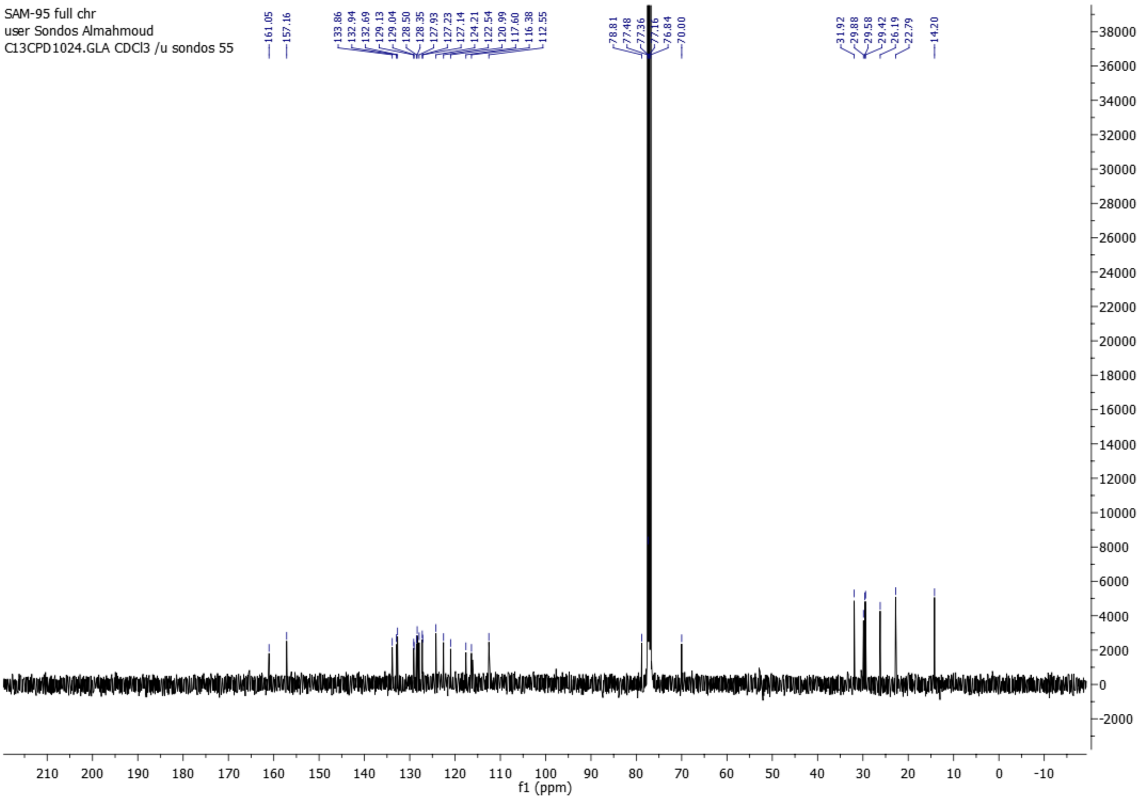

^{1}H NMR of compound 3c

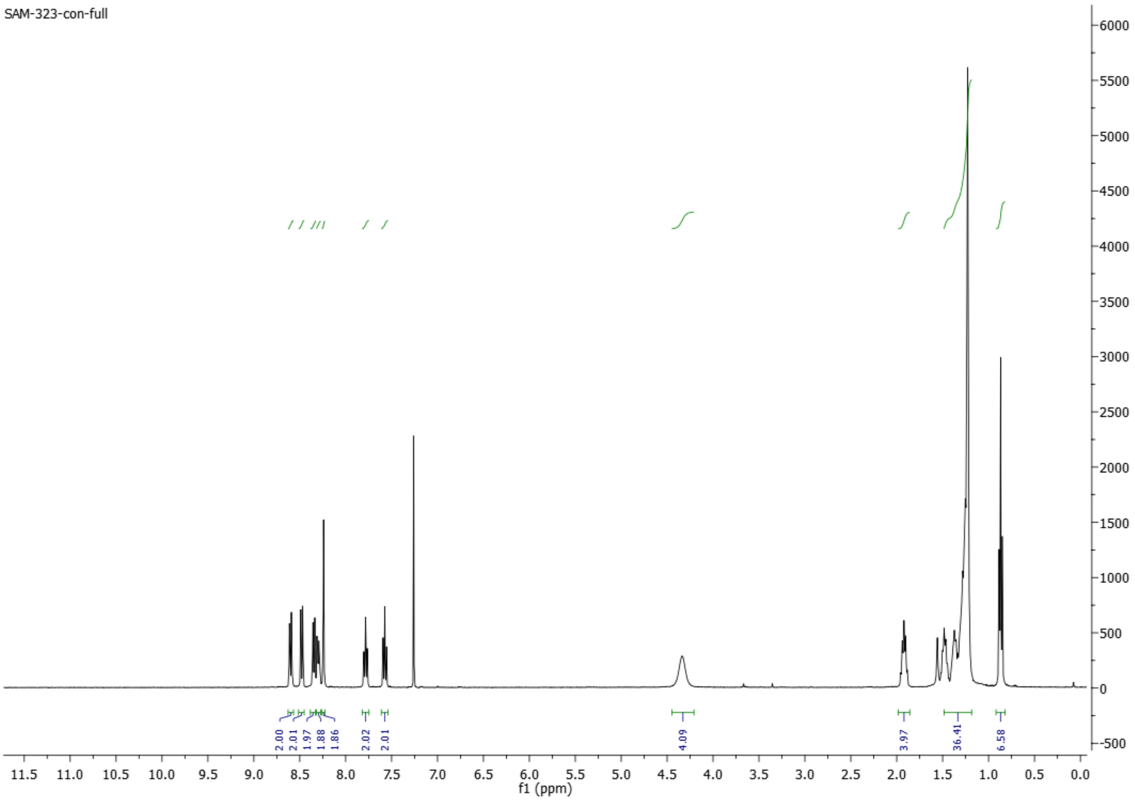

^{13}C NMR of compound 3c

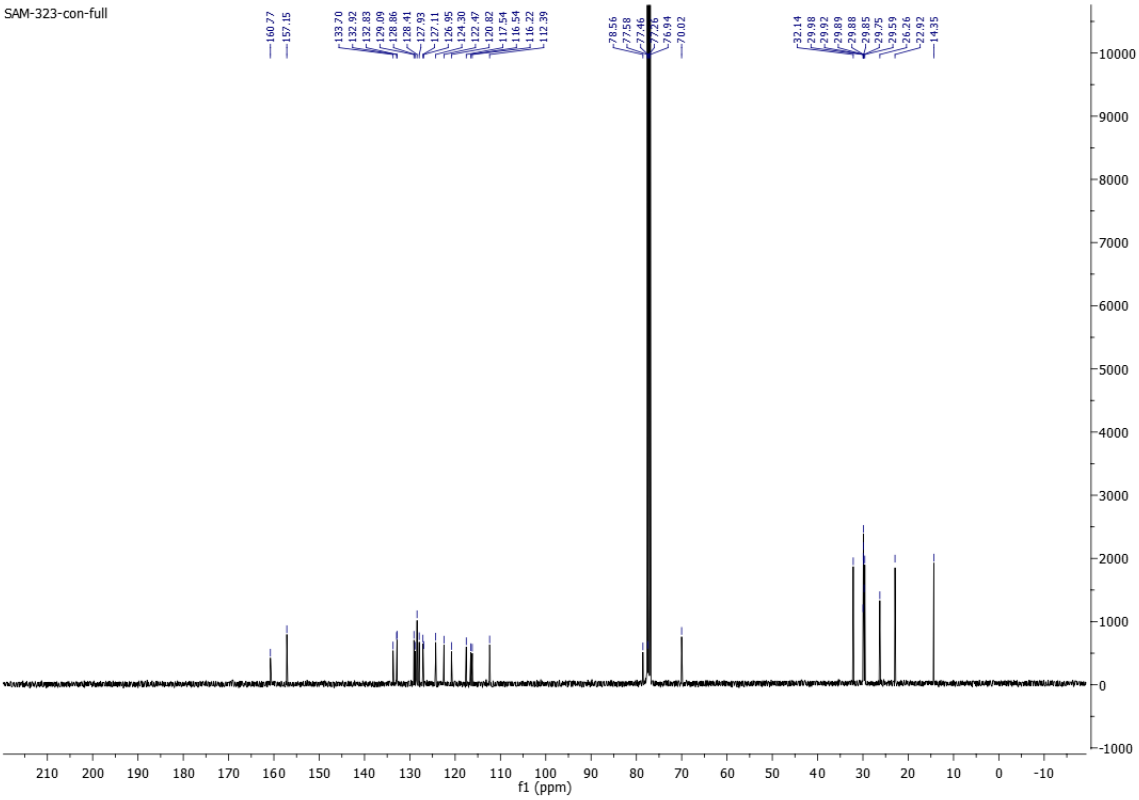

3 Crystallographic studies

Single crystal X-ray diffraction data for **3b** were collected by the EPSRC National Crystallography Service using a ROD, Synergy Custom system, HyPix diffractometer with Cu Kα radiation, λ = 1.54178 Å. Data were collected and processed using CrysAlis PRO 1.171.39.30d (Rigaku OD, 2015). The structure was solved using SHELXT 2018/2 (Sheldrick, 2015a) and refined using *SHELXL* 2018/3 (Sheldrick, 2015b) within Olex2 1.3 (Dolomanov et al., 2009). Non-H atoms were refined with anisotropic atomic displacement parameters (ADPs) and H-atoms were placed in geometrically calculated positions and included as part of a riding model except the Me H-atoms which were included as a rigid rotor.

$C_{56}H_{48}N_4O_2$, M_r = 808.98, Triclinic, *a* = 10.8068 (8), *b* = 12.8088 (6) , *c* = 16.3148 (14) Å, α = 93.100 (6), β = 104.721 (7), γ = 103.717 (5)°, *V* = 2106.1 (3) Å^3, *T* = 100 K, space group *P*-1 (no. 2), Z = 2, brown, plate-like crystal 0.23 × 0.12 × 0.01 mm. 26881 reflections measured, 7857 unique (R_{int} = 0.099), which were used in all calculations. The final $R[F^2 > 2\sigma(F^2)$ 4956 reflections] = 0.125, $wR(F^2)$ = 0.398, 561 parameters.

CCDC 2128169 contains the supplementary crystallographic data for this paper. These data can be obtained free of charge from The Cambridge Crystallographic Data Centre via www.ccdc.cam.ac.uk/structure

Dolomanov, O. V., Bourhis, L. J., Gildea, R. J., Howard, J. A. K. & Puschmann, H. (2009). *J. Appl. Cryst.* **42**, 339–341.

Sheldrick, G. M. (2015a). *Acta Cryst.* A**71**, 3–8.

Sheldrick, G. M. (2015b). *Acta Cryst.* C**71**, 3–8.

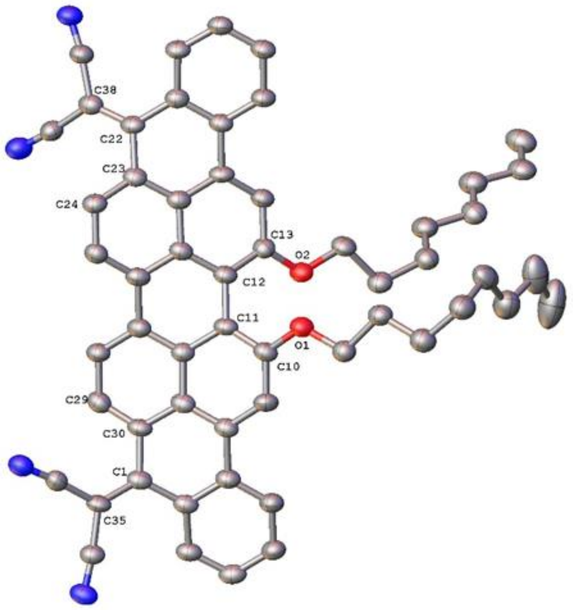

Figure S1: View of the crystal structure of **3b**, displacement ellipsoids drawn at 50% probability level, H-atoms omitted for clarity. Selected atom labels given for torsion angles in Table S1.

Table S1: Selected torsion angles for **3b** and comparison with calculated values for **Va-CN** (OMe substituent) from Zhu et al.[2]

Torsion angle (°)	Experimental crystal structure **3b**	Va-CN Zhu et al
C10—C11—C12—C13	33.3 (8)	33.57
C11—C12—C13—O2	13.1 (8)	11.14
O1—C10—C11—C12	15.1 (8)	11.12
C25—C26—C27—C28	19.4 (8)	12.04
C35—C1—C30—C29	-26.9 (8)	34.23
C38—C22—C23—C24	-29.0 (7)	34.21

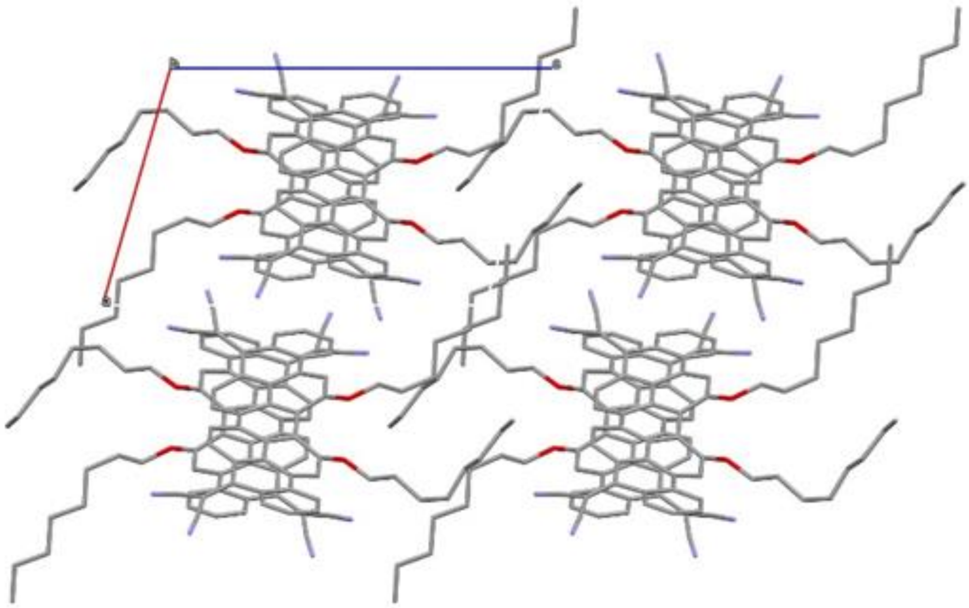

Figure S2: Packing diagram of the structure of **3b**, viewed along the *b*-axis, showing the stacks of molecules linked by π–π interactions and the alternating aromatic and aliphatic layers.

[2] B. Liu, D. Fan, Q. Zhang, Y. Chen and W. Zhu, Frontiers of Chemistry in China, 2010, 5, 200-207.

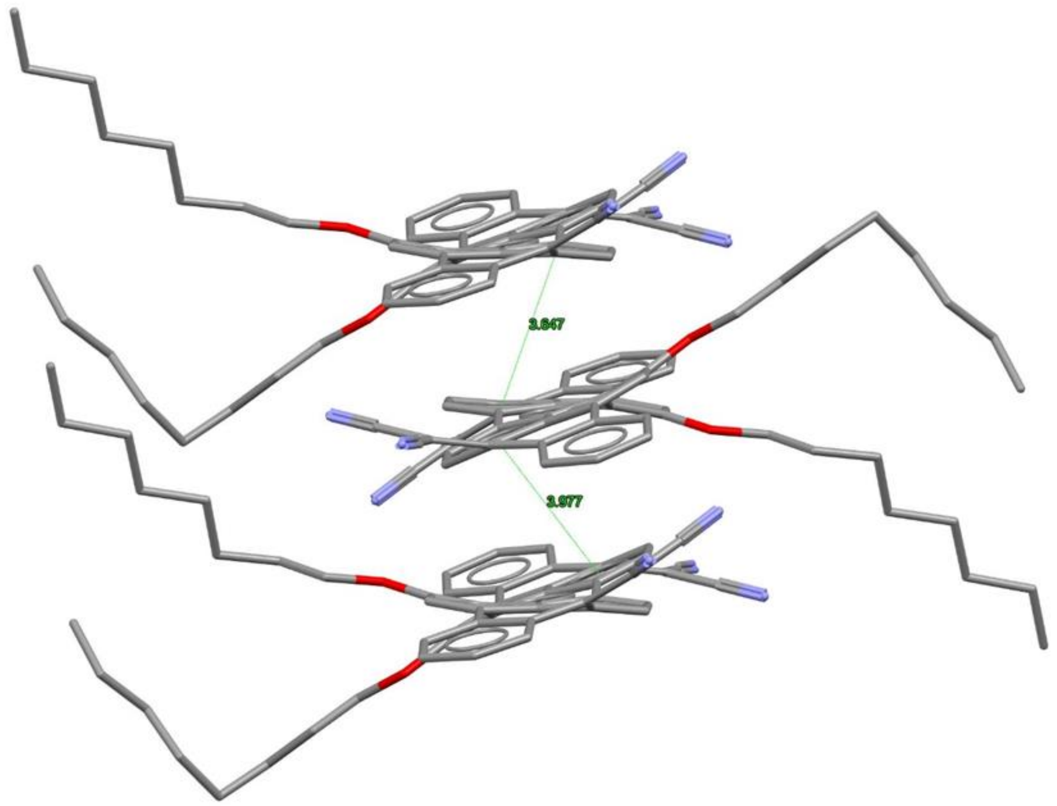

Figure S3: View showing π–π stacking in **3b**; two distinct π–π interactions between parallel rings with centroid–centroid distances of 3.65 and 3.98 Å.

Crystal data

$C_{56}H_{48}N_4O_2$	$Z = 2$
$M_r = 808.98$	$F(000) = 856$
Triclinic, $P\bar{1}$	$D_x = 1.276$ Mg m^{-3}
$a = 10.8068$ (8) Å	Cu $K\alpha$ radiation, $\lambda = 1.54178$ Å
$b = 12.8088$ (6) Å	Cell parameters from 6908 reflections
$c = 16.3148$ (14) Å	$\theta = 2.8–75.3°$
$\alpha = 93.100$ (6)°	$\mu = 0.61$ mm^{-1}
$\beta = 104.721$ (7)°	$T = 100$ K
$\gamma = 103.717$ (5)°	Plate, brown
$V = 2106.1$ (3) Å^3	0.23 × 0.12 × 0.01 mm

Data collection

ROD, Synergy Custom system, HyPix diffractometer	7857 independent reflections
Radiation source: Rotating-anode X-ray tube, Rigaku (Cu) X-ray Source	4956 reflections with $I > 2\sigma(I)$
Mirror monochromator	R_{int} = 0.099
Detector resolution: 10.0000 pixels mm^{-1}	θ_{max} = 76.2°, θ_{min} = 3.6°
ω scans	$h = -13 \rightarrow 13$
Absorption correction: multi-scan *CrysAlis PRO* 1.171.41.112a (Rigaku Oxford Diffraction, 2021) Empirical absorption correction using spherical harmonics, implemented in SCALE3 ABSPACK scaling algorithm.	$k = -11 \rightarrow 15$
T_{min} = 0.635, T_{max} = 1.000	$l = -20 \rightarrow 20$
26881 measured reflections	

Refinement

Refinement on F^2	0 restraints
Least-squares matrix: full	Hydrogen site location: inferred from neighbouring sites
$R[F^2 > 2\sigma(F^2)]$ = 0.125	H-atom parameters constrained
$wR(F^2)$ = 0.398	$w = 1/[\sigma^2(F_o^2) + (0.2P)^2 + 5.P]$ where $P = (F_o^2 + 2F_c^2)/3$
S = 1.09	$(\Delta/\sigma)_{max}$ < 0.001
7857 reflections	$\Delta\rangle_{max}$ = 0.57 e Å^{-3}
561 parameters	$\Delta\rangle_{min}$ = -0.43 e Å^{-3}

Special details

Geometry. All esds (except the esd in the dihedral angle between two l.s. planes) are estimated using the full covariance matrix. The cell esds are taken into account individually in the estimation of esds in distances, angles and torsion angles; correlations between esds in cell parameters are only used when they are defined by crystal symmetry. An approximate (isotropic) treatment of cell esds is used for estimating esds involving l.s. planes.

Fractional atomic coordinates and isotropic or equivalent isotropic displacement parameters (Å^2) for (2021ncs0324z)

	x	y	z	U_{iso}*/U_{eq}
C1	0.2468 (5)	1.0142 (4)	0.5277 (4)	0.0390 (13)
C2	0.2096 (5)	1.0535 (4)	0.4439 (4)	0.0377 (12)
C3	0.1619 (5)	1.1469 (4)	0.4340 (4)	0.0405 (13)
H3	0.157646	1.188312	0.482850	0.049*
C4	0.1215 (5)	1.1786 (4)	0.3542 (4)	0.0437 (14)
H4	0.087782	1.240714	0.348458	0.052*
C5	0.1294 (6)	1.1214 (5)	0.2833 (4)	0.0446 (14)
H5	0.101610	1.144063	0.228662	0.053*
C6	0.1777 (6)	1.0309 (5)	0.2911 (4)	0.0432 (13)
H6	0.181950	0.991221	0.241406	0.052*
C7	0.2207 (5)	0.9963 (4)	0.3711 (4)	0.0402 (13)
C8	0.2806 (5)	0.9042 (4)	0.3799 (4)	0.0368 (12)
C9	0.2790 (5)	0.8367 (4)	0.3100 (4)	0.0407 (13)
H9	0.225215	0.841590	0.255086	0.049*
C10	0.3566 (5)	0.7600 (4)	0.3189 (4)	0.0392 (12)
C11	0.4423 (5)	0.7568 (4)	0.3978 (4)	0.0370 (12)
C12	0.5445 (5)	0.6965 (4)	0.4101 (4)	0.0367 (12)
C13	0.6138 (6)	0.6815 (4)	0.3511 (4)	0.0385 (12)
C14	0.6935 (5)	0.6086 (4)	0.3620 (4)	0.0382 (12)
H14	0.734870	0.595226	0.319110	0.046*
C15	0.7129 (5)	0.5557 (4)	0.4344 (4)	0.0375 (12)
C16	0.7789 (5)	0.4679 (4)	0.4421 (4)	0.0377 (12)
C17	0.7949 (5)	0.4148 (4)	0.3696 (4)	0.0402 (13)
H17	0.766873	0.438965	0.315588	0.048*
C18	0.8515 (6)	0.3269 (4)	0.3755 (4)	0.0424 (13)
H18	0.862853	0.292220	0.325937	0.051*
C19	0.8906 (6)	0.2910 (4)	0.4539 (4)	0.0424 (13)
H19	0.928465	0.231034	0.458100	0.051*
C20	0.8755 (5)	0.3411 (4)	0.5261 (4)	0.0400 (13)
H20	0.903138	0.315219	0.579370	0.048*
C21	0.8195 (5)	0.4305 (4)	0.5224 (4)	0.0380 (12)
C22	0.8026 (5)	0.4858 (4)	0.5980 (4)	0.0380 (12)
C23	0.7019 (5)	0.5484 (4)	0.5829 (4)	0.0375 (12)
C24	0.6424 (5)	0.5710 (4)	0.6458 (4)	0.0401 (13)
H24	0.653695	0.535366	0.695765	0.048*
C25	0.5657 (5)	0.6464 (4)	0.6354 (4)	0.0394 (12)

H25	0.529476	0.664412	0.679897	0.047*
C26	0.5424 (5)	0.6948 (4)	0.5610 (4)	0.0381 (12)
C27	0.4819 (5)	0.7862 (4)	0.5531 (4)	0.0386 (12)
C28	0.4803 (5)	0.8497 (4)	0.6245 (4)	0.0393 (12)
H28	0.526406	0.838424	0.679782	0.047*
C29	0.4113 (6)	0.9303 (4)	0.6158 (4)	0.0421 (13)
H29	0.415242	0.975733	0.664948	0.050*
C30	0.3383 (5)	0.9442 (4)	0.5371 (4)	0.0374 (12)
C31	0.3476 (5)	0.8878 (4)	0.4627 (4)	0.0386 (12)
C32	0.4256 (5)	0.8107 (4)	0.4709 (4)	0.0367 (12)
C33	0.5813 (5)	0.6579 (4)	0.4903 (4)	0.0363 (12)
C34	0.6654 (5)	0.5851 (4)	0.5029 (4)	0.0381 (12)
C35	0.1907 (5)	1.0312 (4)	0.5923 (4)	0.0385 (12)
C36	0.1059 (5)	1.1023 (4)	0.5940 (4)	0.0406 (13)
C37	0.2013 (5)	0.9744 (4)	0.6655 (4)	0.0399 (12)
C38	0.8810 (6)	0.4904 (4)	0.6799 (4)	0.0411 (13)
C39	0.8887 (6)	0.5661 (5)	0.7498 (4)	0.0426 (13)
C40	0.9777 (6)	0.4295 (4)	0.7065 (4)	0.0416 (13)
C41	0.2652 (6)	0.6901 (4)	0.1709 (4)	0.0434 (13)
H41A	0.294230	0.762126	0.152533	0.052*
H41B	0.171861	0.678868	0.172257	0.052*
C42	0.2759 (6)	0.6025 (5)	0.1083 (4)	0.0455 (14)
H42A	0.366809	0.619064	0.101678	0.055*
H42B	0.258215	0.531807	0.130783	0.055*
C43	0.1777 (7)	0.5960 (5)	0.0227 (5)	0.0533 (16)
H43A	0.193735	0.668202	0.002501	0.064*
H43B	0.087281	0.578071	0.030249	0.064*
C44	0.1835 (8)	0.5125 (6)	-0.0462 (5)	0.0593 (17)
H44A	0.167264	0.440407	-0.025739	0.071*
H44B	0.110515	0.509449	-0.097935	0.071*
C45	0.3106 (8)	0.5333 (6)	-0.0707 (5)	0.0609 (18)
H45A	0.302453	0.476175	-0.116578	0.073*
H45B	0.382243	0.527562	-0.020797	0.073*
C46	0.3500 (9)	0.6423 (7)	-0.1010 (6)	0.072 (2)
H46A	0.369938	0.699485	-0.052718	0.087*
H46B	0.273957	0.652093	-0.145875	0.087*
C47	0.4700 (10)	0.6574 (9)	-0.1367 (6)	0.088 (3)
H47A	0.544660	0.641828	-0.094031	0.106*
H47B	0.447909	0.605608	-0.188756	0.106*
C48	0.5114 (14)	0.7724 (12)	-0.1580 (8)	0.121 (5)

H48A	0.443241	0.783914	-0.206637	0.182*
H48B	0.595667	0.783461	-0.172767	0.182*
H48C	0.522002	0.823870	-0.108522	0.182*
C49	0.6645 (6)	0.7228 (4)	0.2195 (4)	0.0439 (13)
H49A	0.626491	0.647106	0.192076	0.053*
H49B	0.761285	0.734669	0.243287	0.053*
C50	0.6358 (6)	0.8009 (4)	0.1547 (4)	0.0456 (14)
H50A	0.672835	0.876082	0.183329	0.055*
H50B	0.538770	0.788751	0.132188	0.055*
C51	0.6949 (6)	0.7870 (5)	0.0806 (4)	0.0472 (14)
H51A	0.658263	0.828525	0.035253	0.057*
H51B	0.665426	0.709645	0.056776	0.057*
C52	0.8463 (7)	0.8230 (5)	0.1025 (5)	0.0517 (15)
H52A	0.877861	0.895963	0.135431	0.062*
H52B	0.883655	0.772590	0.139122	0.062*
C53	0.8971 (7)	0.8261 (5)	0.0235 (5)	0.0516 (15)
H53A	0.856957	0.874588	-0.013916	0.062*
H53B	0.867360	0.752609	-0.008390	0.062*
C54	1.0479 (7)	0.8652 (5)	0.0434 (5)	0.0577 (17)
H54A	1.077846	0.937180	0.077761	0.069*
H54B	1.087707	0.814714	0.078615	0.069*
C55	1.0996 (7)	0.8737 (5)	-0.0352 (5)	0.0547 (17)
H55A	1.057947	0.922471	-0.071417	0.066*
H55B	1.072343	0.801246	-0.068728	0.066*
C56	1.2496 (8)	0.9159 (6)	-0.0145 (6)	0.065 (2)
H56A	1.277741	0.987355	0.019141	0.097*
H56B	1.275767	0.921756	-0.067698	0.097*
H56C	1.291801	0.865736	0.018374	0.097*
N1	0.0383 (5)	1.1569 (4)	0.6018 (4)	0.0482 (13)
N2	0.2019 (5)	0.9309 (4)	0.7265 (4)	0.0484 (12)
N3	0.9061 (6)	0.6259 (5)	0.8094 (4)	0.0563 (14)
N4	1.0562 (5)	0.3858 (4)	0.7357 (4)	0.0486 (12)
O1	0.3462 (4)	0.6859 (3)	0.2538 (3)	0.0441 (10)
O2	0.6047 (4)	0.7433 (3)	0.2862 (3)	0.0421 (9)

Atomic displacement parameters (Å^2) for (2021ncs0324z)

	U^{11}	U^{22}	U^{33}	U^{12}	U^{13}	U^{23}
C1	0.036 (3)	0.024 (2)	0.057 (4)	0.0056 (19)	0.016 (3)	0.004 (2)
C2	0.033 (2)	0.024 (2)	0.058 (4)	0.0055 (18)	0.017 (2)	0.005 (2)

C3	0.036 (3)	0.027 (2)	0.061 (4)	0.009 (2)	0.018 (3)	0.007 (2)
C4	0.038 (3)	0.029 (2)	0.068 (4)	0.009 (2)	0.018 (3)	0.013 (3)
C5	0.048 (3)	0.039 (3)	0.053 (4)	0.018 (2)	0.017 (3)	0.014 (3)
C6	0.045 (3)	0.036 (3)	0.053 (4)	0.016 (2)	0.016 (3)	0.009 (2)
C7	0.040 (3)	0.028 (2)	0.055 (4)	0.010 (2)	0.016 (3)	0.007 (2)
C8	0.035 (2)	0.024 (2)	0.054 (4)	0.0056 (18)	0.018 (2)	0.006 (2)
C9	0.036 (3)	0.034 (3)	0.054 (4)	0.014 (2)	0.012 (3)	0.007 (2)
C10	0.039 (3)	0.028 (2)	0.053 (4)	0.009 (2)	0.018 (3)	0.004 (2)
C11	0.036 (3)	0.025 (2)	0.054 (4)	0.0110 (19)	0.017 (2)	0.008 (2)
C12	0.037 (3)	0.025 (2)	0.051 (3)	0.0096 (19)	0.016 (2)	0.005 (2)
C13	0.045 (3)	0.027 (2)	0.045 (3)	0.009 (2)	0.015 (3)	0.007 (2)
C14	0.040 (3)	0.030 (2)	0.049 (3)	0.012 (2)	0.016 (3)	0.005 (2)
C15	0.035 (2)	0.023 (2)	0.058 (4)	0.0106 (19)	0.015 (3)	0.009 (2)
C16	0.038 (3)	0.024 (2)	0.054 (4)	0.0090 (19)	0.018 (3)	0.007 (2)
C17	0.042 (3)	0.029 (2)	0.053 (4)	0.013 (2)	0.014 (3)	0.006 (2)
C18	0.045 (3)	0.030 (2)	0.058 (4)	0.014 (2)	0.020 (3)	0.007 (2)
C19	0.039 (3)	0.034 (3)	0.059 (4)	0.014 (2)	0.018 (3)	0.010 (3)
C20	0.041 (3)	0.025 (2)	0.057 (4)	0.011 (2)	0.016 (3)	0.009 (2)
C21	0.034 (2)	0.029 (2)	0.052 (4)	0.0074 (19)	0.014 (2)	0.007 (2)
C22	0.038 (3)	0.025 (2)	0.056 (4)	0.0103 (19)	0.019 (3)	0.012 (2)
C23	0.038 (3)	0.023 (2)	0.052 (3)	0.0063 (19)	0.016 (2)	0.006 (2)
C24	0.041 (3)	0.028 (2)	0.054 (4)	0.010 (2)	0.015 (3)	0.009 (2)
C25	0.039 (3)	0.033 (3)	0.050 (4)	0.010 (2)	0.018 (3)	0.006 (2)
C26	0.032 (2)	0.029 (2)	0.054 (4)	0.0088 (19)	0.013 (2)	0.004 (2)
C27	0.037 (3)	0.030 (2)	0.053 (4)	0.010 (2)	0.018 (3)	0.009 (2)
C28	0.041 (3)	0.029 (2)	0.051 (4)	0.012 (2)	0.016 (3)	0.008 (2)
C29	0.045 (3)	0.032 (3)	0.053 (4)	0.008 (2)	0.022 (3)	0.004 (2)
C30	0.036 (3)	0.024 (2)	0.057 (4)	0.0098 (19)	0.019 (3)	0.007 (2)
C31	0.039 (3)	0.024 (2)	0.056 (4)	0.0061 (19)	0.020 (3)	0.007 (2)
C32	0.034 (2)	0.026 (2)	0.050 (3)	0.0066 (19)	0.013 (2)	0.006 (2)
C33	0.032 (2)	0.027 (2)	0.050 (3)	0.0066 (19)	0.014 (2)	0.003 (2)
C34	0.034 (2)	0.027 (2)	0.052 (4)	0.0076 (19)	0.012 (2)	0.005 (2)
C35	0.040 (3)	0.024 (2)	0.057 (4)	0.012 (2)	0.019 (3)	0.005 (2)
C36	0.040 (3)	0.028 (2)	0.055 (4)	0.009 (2)	0.016 (3)	0.007 (2)
C37	0.038 (3)	0.039 (3)	0.044 (3)	0.011 (2)	0.014 (2)	0.003 (2)
C38	0.041 (3)	0.031 (3)	0.056 (4)	0.010 (2)	0.020 (3)	0.011 (2)
C39	0.045 (3)	0.037 (3)	0.047 (4)	0.014 (2)	0.010 (3)	0.009 (3)
C40	0.044 (3)	0.034 (3)	0.052 (4)	0.015 (2)	0.016 (3)	0.006 (2)
C41	0.046 (3)	0.036 (3)	0.050 (4)	0.011 (2)	0.016 (3)	0.008 (2)
C42	0.049 (3)	0.037 (3)	0.055 (4)	0.015 (2)	0.018 (3)	0.011 (3)

C43	0.055 (4)	0.045 (3)	0.062 (4)	0.014 (3)	0.019 (3)	0.007 (3)
C44	0.068 (4)	0.048 (3)	0.060 (4)	0.018 (3)	0.014 (4)	0.004 (3)
C45	0.062 (4)	0.062 (4)	0.058 (5)	0.021 (3)	0.014 (4)	-0.007 (3)
C46	0.073 (5)	0.082 (5)	0.067 (5)	0.025 (4)	0.023 (4)	0.016 (4)
C47	0.071 (5)	0.116 (7)	0.060 (5)	-0.006 (5)	0.018 (4)	-0.004 (5)
C48	0.114 (9)	0.150 (11)	0.068 (7)	-0.022 (8)	0.018 (6)	0.030 (7)
C49	0.051 (3)	0.029 (2)	0.057 (4)	0.014 (2)	0.020 (3)	0.005 (2)
C50	0.051 (3)	0.031 (3)	0.057 (4)	0.011 (2)	0.017 (3)	0.010 (2)
C51	0.055 (3)	0.038 (3)	0.053 (4)	0.015 (2)	0.019 (3)	0.009 (3)
C52	0.058 (4)	0.043 (3)	0.064 (4)	0.022 (3)	0.024 (3)	0.016 (3)
C53	0.063 (4)	0.037 (3)	0.061 (4)	0.014 (3)	0.025 (3)	0.012 (3)
C54	0.060 (4)	0.047 (3)	0.074 (5)	0.021 (3)	0.026 (4)	0.017 (3)
C55	0.062 (4)	0.039 (3)	0.074 (5)	0.021 (3)	0.029 (4)	0.014 (3)
C56	0.070 (4)	0.047 (3)	0.091 (6)	0.020 (3)	0.041 (4)	0.015 (4)
N1	0.049 (3)	0.032 (2)	0.070 (4)	0.012 (2)	0.027 (3)	0.007 (2)
N2	0.050 (3)	0.043 (3)	0.058 (4)	0.017 (2)	0.021 (3)	0.006 (2)
N3	0.058 (3)	0.052 (3)	0.062 (4)	0.024 (3)	0.013 (3)	0.008 (3)
N4	0.052 (3)	0.041 (3)	0.059 (3)	0.021 (2)	0.017 (3)	0.007 (2)
O1	0.049 (2)	0.0372 (19)	0.049 (3)	0.0170 (16)	0.0128 (19)	0.0032 (17)
O2	0.049 (2)	0.0348 (19)	0.051 (2)	0.0173 (16)	0.0204 (19)	0.0122 (17)

Geometric parameters (Å, º) for (2021ncs0324z)

C1—C2	1.475 (9)	C33—C34	1.438 (7)
C1—C30	1.471 (7)	C35—C36	1.440 (7)
C1—C35	1.375 (9)	C35—C37	1.425 (9)
C2—C3	1.411 (7)	C36—N1	1.148 (7)
C2—C7	1.408 (9)	C37—N2	1.166 (8)
C3—H3	0.9500	C38—C39	1.429 (9)
C3—C4	1.380 (9)	C38—C40	1.442 (8)
C4—H4	0.9500	C39—N3	1.151 (9)
C4—C5	1.368 (10)	C40—N4	1.145 (8)
C5—H5	0.9500	C41—H41A	0.9900
C5—C6	1.380 (8)	C41—H41B	0.9900
C6—H6	0.9500	C41—C42	1.521 (9)
C6—C7	1.402 (9)	C41—O1	1.424 (8)
C7—C8	1.471 (7)	C42—H42A	0.9900
C8—C9	1.388 (9)	C42—H42B	0.9900
C8—C31	1.415 (9)	C42—C43	1.508 (10)
C9—H9	0.9500	C43—H43A	0.9900

C9—C10	1.426 (7)	C43—H43B	0.9900
C10—C11	1.391 (9)	C43—C44	1.533 (10)
C10—O1	1.350 (7)	C44—H44A	0.9900
C11—C12	1.473 (7)	C44—H44B	0.9900
C11—C32	1.417 (9)	C44—C45	1.495 (11)
C12—C13	1.392 (8)	C45—H45A	0.9900
C12—C33	1.418 (9)	C45—H45B	0.9900
C13—C14	1.401 (7)	C45—C46	1.509 (12)
C13—O2	1.355 (7)	C46—H46A	0.9900
C14—H14	0.9500	C46—H46B	0.9900
C14—C15	1.388 (8)	C46—C47	1.527 (13)
C15—C16	1.463 (7)	C47—H47A	0.9900
C15—C34	1.411 (9)	C47—H47B	0.9900
C16—C17	1.401 (9)	C47—C48	1.521 (17)
C16—C21	1.419 (9)	C48—H48A	0.9800
C17—H17	0.9500	C48—H48B	0.9800
C17—C18	1.400 (7)	C48—H48C	0.9800
C18—H18	0.9500	C49—H49A	0.9900
C18—C19	1.382 (9)	C49—H49B	0.9900
C19—H19	0.9500	C49—C50	1.519 (8)
C19—C20	1.375 (9)	C49—O2	1.441 (8)
C20—H20	0.9500	C50—H50A	0.9900
C20—C21	1.413 (7)	C50—H50B	0.9900
C21—C22	1.462 (9)	C50—C51	1.524 (9)
C22—C23	1.479 (7)	C51—H51A	0.9900
C22—C38	1.378 (9)	C51—H51B	0.9900
C23—C24	1.394 (9)	C51—C52	1.531 (9)
C23—C34	1.405 (9)	C52—H52A	0.9900
C24—H24	0.9500	C52—H52B	0.9900
C24—C25	1.405 (8)	C52—C53	1.522 (10)
C25—H25	0.9500	C53—H53A	0.9900
C25—C26	1.386 (8)	C53—H53B	0.9900
C26—C27	1.466 (7)	C53—C54	1.530 (10)
C26—C33	1.418 (9)	C54—H54A	0.9900
C27—C28	1.390 (9)	C54—H54B	0.9900
C27—C32	1.412 (9)	C54—C55	1.523 (11)
C28—H28	0.9500	C55—H55A	0.9900
C28—C29	1.404 (8)	C55—H55B	0.9900
C29—H29	0.9500	C55—C56	1.523 (10)
C29—C30	1.371 (9)	C56—H56A	0.9800

C30—C31	1.416 (9)	C56—H56B	0.9800
C31—C32	1.434 (7)	C56—H56C	0.9800
C30—C1—C2	116.7 (5)	N1—C36—C35	174.6 (7)
C35—C1—C2	123.2 (5)	N2—C37—C35	174.7 (6)
C35—C1—C30	119.8 (5)	C22—C38—C39	124.2 (5)
C3—C2—C1	122.0 (5)	C22—C38—C40	126.0 (5)
C3—C2—C7	118.6 (6)	C39—C38—C40	109.5 (5)
C7—C2—C1	119.5 (5)	N3—C39—C38	173.8 (6)
C2—C3—H3	119.7	N4—C40—C38	173.1 (7)
C4—C3—C2	120.7 (6)	H41A—C41—H41B	108.3
C4—C3—H3	119.7	C42—C41—H41A	109.8
C3—C4—H4	119.7	C42—C41—H41B	109.8
C5—C4—C3	120.6 (5)	O1—C41—H41A	109.8
C5—C4—H4	119.7	O1—C41—H41B	109.8
C4—C5—H5	120.0	O1—C41—C42	109.3 (5)
C4—C5—C6	120.0 (6)	C41—C42—H42A	109.6
C6—C5—H5	120.0	C41—C42—H42B	109.6
C5—C6—H6	119.4	H42A—C42—H42B	108.1
C5—C6—C7	121.1 (6)	C43—C42—C41	110.2 (5)
C7—C6—H6	119.4	C43—C42—H42A	109.6
C2—C7—C8	120.1 (6)	C43—C42—H42B	109.6
C6—C7—C2	118.9 (5)	C42—C43—H43A	108.6
C6—C7—C8	121.0 (6)	C42—C43—H43B	108.6
C9—C8—C7	122.5 (6)	C42—C43—C44	114.5 (6)
C9—C8—C31	119.2 (5)	H43A—C43—H43B	107.6
C31—C8—C7	118.2 (5)	C44—C43—H43A	108.6
C8—C9—H9	119.4	C44—C43—H43B	108.6
C8—C9—C10	121.2 (6)	C43—C44—H44A	108.4
C10—C9—H9	119.4	C43—C44—H44B	108.4
C11—C10—C9	119.9 (5)	H44A—C44—H44B	107.5
O1—C10—C9	122.2 (6)	C45—C44—C43	115.5 (6)
O1—C10—C11	117.8 (5)	C45—C44—H44A	108.4
C10—C11—C12	123.8 (5)	C45—C44—H44B	108.4
C10—C11—C32	117.8 (5)	C44—C45—H45A	108.7
C32—C11—C12	118.3 (5)	C44—C45—H45B	108.7
C13—C12—C11	124.6 (5)	C44—C45—C46	114.3 (6)
C13—C12—C33	118.0 (5)	H45A—C45—H45B	107.6
C33—C12—C11	117.3 (5)	C46—C45—H45A	108.7
C12—C13—C14	120.4 (5)	C46—C45—H45B	108.7

O2—C13—C12	117.0 (5)	C45—C46—H46A	108.8
O2—C13—C14	122.5 (5)	C45—C46—H46B	108.8
C13—C14—H14	119.4	C45—C46—C47	113.9 (8)
C15—C14—C13	121.2 (5)	H46A—C46—H46B	107.7
C15—C14—H14	119.4	C47—C46—H46A	108.8
C14—C15—C16	122.4 (5)	C47—C46—H46B	108.8
C14—C15—C34	119.6 (5)	C46—C47—H47A	109.5
C34—C15—C16	118.0 (5)	C46—C47—H47B	109.5
C17—C16—C15	120.5 (5)	H47A—C47—H47B	108.1
C17—C16—C21	119.1 (5)	C48—C47—C46	110.8 (10)
C21—C16—C15	120.2 (5)	C48—C47—H47A	109.5
C16—C17—H17	119.4	C48—C47—H47B	109.5
C18—C17—C16	121.1 (6)	C47—C48—H48A	109.5
C18—C17—H17	119.4	C47—C48—H48B	109.5
C17—C18—H18	120.3	C47—C48—H48C	109.5
C19—C18—C17	119.4 (6)	H48A—C48—H48B	109.5
C19—C18—H18	120.3	H48A—C48—H48C	109.5
C18—C19—H19	119.6	H48B—C48—H48C	109.5
C20—C19—C18	120.7 (5)	H49A—C49—H49B	108.5
C20—C19—H19	119.6	C50—C49—H49A	110.3
C19—C20—H20	119.4	C50—C49—H49B	110.3
C19—C20—C21	121.3 (6)	O2—C49—H49A	110.3
C21—C20—H20	119.4	O2—C49—H49B	110.3
C16—C21—C22	119.1 (5)	O2—C49—C50	107.2 (4)
C20—C21—C16	118.4 (5)	C49—C50—H50A	109.2
C20—C21—C22	122.6 (5)	C49—C50—H50B	109.2
C21—C22—C23	116.5 (5)	C49—C50—C51	112.1 (5)
C38—C22—C21	124.3 (5)	H50A—C50—H50B	107.9
C38—C22—C23	119.0 (5)	C51—C50—H50A	109.2
C24—C23—C22	121.5 (5)	C51—C50—H50B	109.2
C24—C23—C34	119.7 (5)	C50—C51—H51A	108.4
C34—C23—C22	118.8 (5)	C50—C51—H51B	108.4
C23—C24—H24	120.0	C50—C51—C52	115.6 (6)
C23—C24—C25	120.0 (5)	H51A—C51—H51B	107.4
C25—C24—H24	120.0	C52—C51—H51A	108.4
C24—C25—H25	119.6	C52—C51—H51B	108.4
C26—C25—C24	120.8 (5)	C51—C52—H52A	109.1
C26—C25—H25	119.6	C51—C52—H52B	109.1
C25—C26—C27	122.2 (5)	H52A—C52—H52B	107.8
C25—C26—C33	119.7 (5)	C53—C52—C51	112.7 (6)

C33—C26—C27	118.0 (5)	C53—C52—H52A	109.1
C28—C27—C26	121.7 (6)	C53—C52—H52B	109.1
C28—C27—C32	119.4 (5)	C52—C53—H53A	108.8
C32—C27—C26	118.9 (5)	C52—C53—H53B	108.8
C27—C28—H28	119.6	C52—C53—C54	113.8 (7)
C27—C28—C29	120.8 (6)	H53A—C53—H53B	107.7
C29—C28—H28	119.6	C54—C53—H53A	108.8
C28—C29—H29	119.7	C54—C53—H53B	108.8
C30—C29—C28	120.6 (6)	C53—C54—H54A	108.7
C30—C29—H29	119.7	C53—C54—H54B	108.7
C29—C30—C1	121.6 (5)	H54A—C54—H54B	107.6
C29—C30—C31	119.7 (5)	C55—C54—C53	114.3 (7)
C31—C30—C1	118.7 (6)	C55—C54—H54A	108.7
C8—C31—C32	118.5 (5)	C55—C54—H54B	108.7
C30—C31—C8	122.0 (5)	C54—C55—H55A	108.8
C30—C31—C32	119.6 (6)	C54—C55—H55B	108.8
C11—C32—C31	121.0 (5)	C54—C55—C56	113.7 (7)
C27—C32—C11	120.2 (5)	H55A—C55—H55B	107.7
C27—C32—C31	118.7 (5)	C56—C55—H55A	108.8
C12—C33—C34	120.4 (5)	C56—C55—H55B	108.8
C26—C33—C12	121.0 (5)	C55—C56—H56A	109.5
C26—C33—C34	118.5 (5)	C55—C56—H56B	109.5
C15—C34—C33	118.4 (5)	C55—C56—H56C	109.5
C23—C34—C15	121.9 (5)	H56A—C56—H56B	109.5
C23—C34—C33	119.7 (5)	H56A—C56—H56C	109.5
C1—C35—C36	125.9 (5)	H56B—C56—H56C	109.5
C1—C35—C37	124.1 (5)	C10—O1—C41	119.5 (4)
C37—C35—C36	109.9 (5)	C13—O2—C49	118.6 (4)
C1—C2—C3—C4	-176.7 (5)	C21—C16—C17—C18	-0.8 (8)
C1—C2—C7—C6	176.3 (5)	C21—C22—C23—C24	156.4 (5)
C1—C2—C7—C8	-5.7 (7)	C21—C22—C23—C34	-23.3 (7)
C1—C30—C31—C8	5.6 (7)	C21—C22—C38—C39	162.7 (5)
C1—C30—C31—C32	-173.6 (4)	C21—C22—C38—C40	-9.8 (8)
C2—C1—C30—C29	159.4 (5)	C22—C23—C24—C25	168.5 (5)
C2—C1—C30—C31	-22.3 (7)	C22—C23—C34—C15	5.9 (8)
C2—C1—C35—C36	-10.5 (9)	C22—C23—C34—C33	-171.8 (5)
C2—C1—C35—C37	164.9 (5)	C23—C22—C38—C39	-11.5 (8)
C2—C3—C4—C5	-1.4 (8)	C23—C22—C38—C40	175.9 (5)
C2—C7—C8—C9	171.6 (5)	C23—C24—C25—C26	3.4 (8)

C2—C7—C8—C31	-11.4 (7)	C24—C23—C34—C15	-173.8 (5)
C3—C2—C7—C6	-3.2 (8)	C24—C23—C34—C33	8.6 (8)
C3—C2—C7—C8	174.7 (5)	C24—C25—C26—C27	-169.8 (5)
C3—C4—C5—C6	0.2 (9)	C24—C25—C26—C33	8.3 (8)
C4—C5—C6—C7	-0.7 (9)	C25—C26—C27—C28	19.4 (8)
C5—C6—C7—C2	2.2 (8)	C25—C26—C27—C32	-161.4 (5)
C5—C6—C7—C8	-175.7 (5)	C25—C26—C33—C12	172.2 (5)
C6—C7—C8—C9	-10.5 (8)	C25—C26—C33—C34	-11.3 (8)
C6—C7—C8—C31	166.5 (5)	C26—C27—C28—C29	-174.1 (5)
C7—C2—C3—C4	2.9 (8)	C26—C27—C32—C11	-7.9 (7)
C7—C8—C9—C10	169.2 (5)	C26—C27—C32—C31	169.8 (5)
C7—C8—C31—C30	11.5 (7)	C26—C33—C34—C15	-174.7 (5)
C7—C8—C31—C32	-169.3 (5)	C26—C33—C34—C23	3.0 (7)
C8—C9—C10—C11	-4.2 (8)	C27—C26—C33—C12	-9.6 (8)
C8—C9—C10—O1	172.9 (5)	C27—C26—C33—C34	166.9 (5)
C8—C31—C32—C11	3.9 (7)	C27—C28—C29—C30	3.5 (8)
C8—C31—C32—C27	-173.8 (5)	C28—C27—C32—C11	171.2 (5)
C9—C8—C31—C30	-171.4 (5)	C28—C27—C32—C31	-11.1 (7)
C9—C8—C31—C32	7.8 (7)	C28—C29—C30—C1	169.1 (5)
C9—C10—C11—C12	-167.6 (5)	C28—C29—C30—C31	-9.2 (8)
C9—C10—C11—C32	15.6 (7)	C29—C30—C31—C8	-176.1 (5)
C9—C10—O1—C41	6.2 (8)	C29—C30—C31—C32	4.7 (7)
C10—C11—C12—C13	33.3 (8)	C30—C1—C2—C3	-158.1 (5)
C10—C11—C12—C33	-151.5 (5)	C30—C1—C2—C7	22.3 (7)
C10—C11—C32—C27	162.1 (5)	C30—C1—C35—C36	176.2 (5)
C10—C11—C32—C31	-15.5 (7)	C30—C1—C35—C37	-8.3 (8)
C11—C10—O1—C41	-176.6 (5)	C30—C31—C32—C11	-176.9 (4)
C11—C12—C13—C14	-169.6 (5)	C30—C31—C32—C27	5.4 (7)
C11—C12—C13—O2	13.1 (8)	C31—C8—C9—C10	-7.8 (8)
C11—C12—C33—C26	-12.8 (7)	C32—C11—C12—C13	-149.9 (5)
C11—C12—C33—C34	170.8 (5)	C32—C11—C12—C33	25.3 (7)
C12—C11—C32—C27	-14.9 (7)	C32—C27—C28—C29	6.8 (8)
C12—C11—C32—C31	167.5 (4)	C33—C12—C13—C14	15.2 (8)
C12—C13—C14—C15	-4.7 (8)	C33—C12—C13—O2	-162.0 (5)
C12—C13—O2—C49	-174.1 (5)	C33—C26—C27—C28	-158.7 (5)
C12—C33—C34—C15	1.7 (7)	C33—C26—C27—C32	20.4 (7)
C12—C33—C34—C23	179.4 (5)	C34—C15—C16—C17	162.1 (5)
C13—C12—C33—C26	162.7 (5)	C34—C15—C16—C21	-13.6 (8)
C13—C12—C33—C34	-13.7 (7)	C34—C23—C24—C25	-11.9 (8)
C13—C14—C15—C16	170.8 (5)	C35—C1—C2—C3	28.5 (8)

C13—C14—C15—C34	-7.8 (8)	C35—C1—C2—C7	-151.1 (5)
C14—C13—O2—C49	8.7 (8)	C35—C1—C30—C29	-26.9 (8)
C14—C15—C16—C17	-16.5 (8)	C35—C1—C30—C31	151.4 (5)
C14—C15—C16—C21	167.8 (5)	C38—C22—C23—C24	-29.0 (7)
C14—C15—C34—C23	-168.7 (5)	C38—C22—C23—C34	151.4 (5)
C14—C15—C34—C33	9.0 (8)	C41—C42—C43—C44	178.1 (5)
C15—C16—C17—C18	-176.6 (5)	C42—C41—O1—C10	177.0 (5)
C15—C16—C21—C20	176.2 (5)	C42—C43—C44—C45	-63.3 (8)
C15—C16—C21—C22	-4.2 (7)	C43—C44—C45—C46	-56.6 (9)
C16—C15—C34—C23	12.7 (8)	C44—C45—C46—C47	-172.6 (7)
C16—C15—C34—C33	-169.6 (5)	C45—C46—C47—C48	-174.7 (8)
C16—C17—C18—C19	0.8 (8)	C49—C50—C51—C52	-69.5 (7)
C16—C21—C22—C23	22.2 (7)	C50—C49—O2—C13	178.4 (5)
C16—C21—C22—C38	-152.1 (5)	C50—C51—C52—C53	-169.6 (5)
C17—C16—C21—C20	0.4 (7)	C51—C52—C53—C54	178.2 (5)
C17—C16—C21—C22	-179.9 (5)	C52—C53—C54—C55	-177.3 (5)
C17—C18—C19—C20	-0.5 (8)	C53—C54—C55—C56	178.2 (5)
C18—C19—C20—C21	0.1 (8)	O1—C10—C11—C12	15.1 (8)
C19—C20—C21—C16	-0.1 (8)	O1—C10—C11—C32	-161.7 (5)
C19—C20—C21—C22	-179.7 (5)	O1—C41—C42—C43	172.8 (5)
C20—C21—C22—C23	-158.1 (5)	O2—C13—C14—C15	172.4 (5)
C20—C21—C22—C38	27.6 (8)	O2—C49—C50—C51	179.9 (5)

Document origin: *publCIF* [Westrip, S. P. (2010). *J. Apply. Cryst.*, **43**, 920-925].

4 OFET Plots

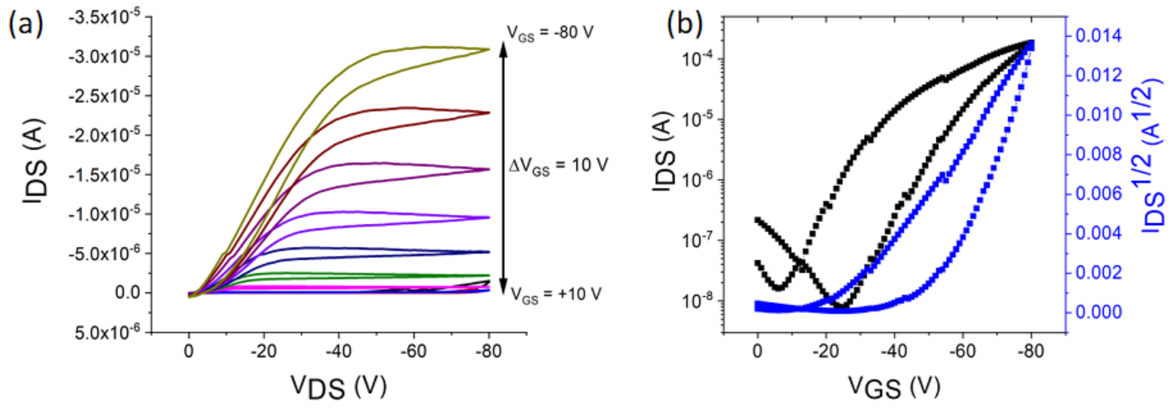

Figure S4: (a) Output and (b) transfer characteristics (V_{DS} = -70 V) of OFET fabricated using **3b** layer. Channel width = 1 cm, channel length = 20 μm

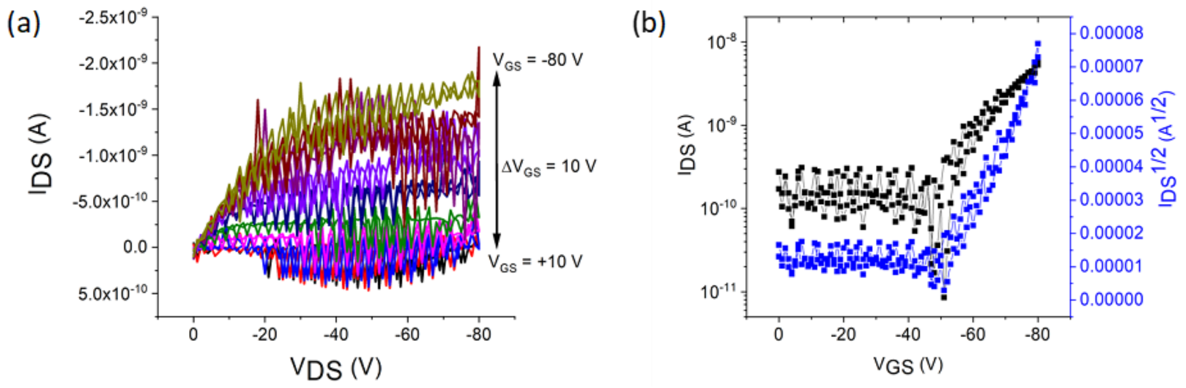

Figure S5: (a) Output and (b) transfer characteristics (V_{DS} = -70 V) of OFET fabricated using **2b** layer. Channel width = 1 cm, channel length = 10 μm.

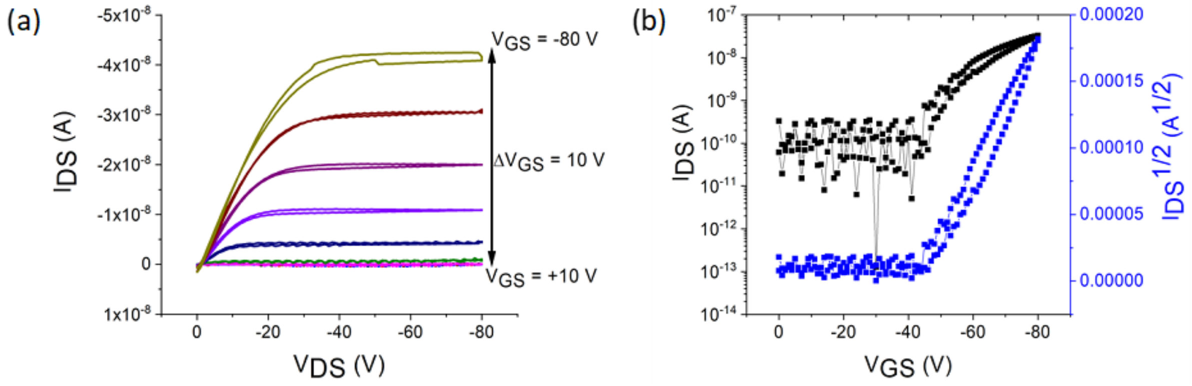

Figure S6: (a) Output and (b) transfer characteristics (V_{DS} = −70 V) of OFET fabricated using **2a** layer. Channel width = 1 cm, channel length = 20 μm

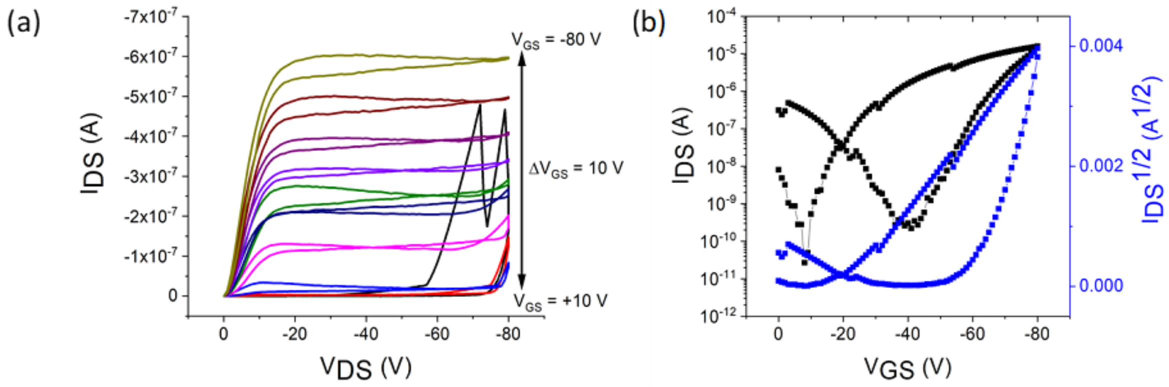

Figure S7: (a) Output and (b) transfer characteristics (V_{DS} = -70 V) of OFET fabricated using **3c** layer. Channel width = 1 cm, channel length = 20 μm

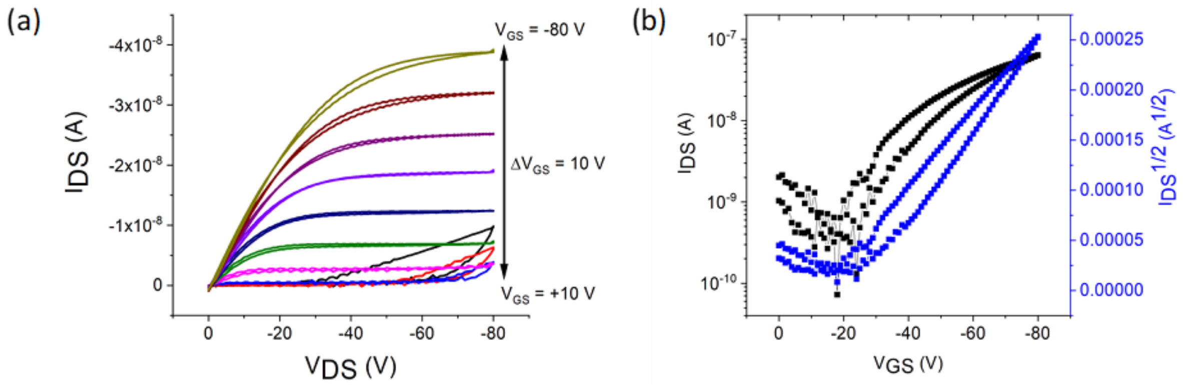

Figure S8: (a) Output and (b) transfer characteristics (V_{DS} = -70 V) of OFET fabricated using **3a** layer. Channel width = 1 cm, channel length = 20 μm
